# Green Fabrication of Sulfonium‐Containing Bismuth Materials for High‐Sensitivity X‐Ray Detection

**DOI:** 10.1002/adma.202418626

**Published:** 2025-04-10

**Authors:** Allan Starkholm, Dominik Al‐Sabbagh, Sema Sarisozen, Alexander von Reppert, Matthias Rössle, Markus Ostermann, Eva Unger, Franziska Emmerling, Lars Kloo, Per H. Svensson, Felix Lang, Olena Maslyanchuk

**Affiliations:** ^1^ Department Solution‐Processing of Hybrid Materials and Devices Helmholtz‐Zentrum Berlin 14109 Berlin Germany; ^2^ Department of Materials Chemistry Federal Institute for Materials Research and Testing 12205 Berlin Germany; ^3^ Freigeist Juniorgroup, Radiation Tolerant Electronics with Soft Semiconductors (ROSI) University of Potsdam 14476 Potsdam‐Golm Germany; ^4^ Soft Matter Physics and Optoelectronics Group University of Potsdam 14476 Potsdam‐Golm Germany; ^5^ Research Group Ultrafast Dynamics Helmholtz‐Zentrum Berlin 14109 Berlin Germany; ^6^ Department of Process Analytical Technology Federal Institute for Materials Research and Testing 12205 Berlin Germany; ^7^ Applied Physical Chemistry, Department of Chemistry KTH Royal Institute of Technology Stockholm SE‐114 28 Sweden

**Keywords:** compressed pellets, heterometallic iodobismuthate, long‐term stability, mechanosynthesis, new materials, sulfonium iodobismuthate, X‐ray detectors

## Abstract

Organic–inorganic hybrid materials based on lead and bismuth have recently been proposed as novel X‐ and gamma‐ray detectors for medical imaging, non‐destructive testing, and security, due to their high atomic numbers and facile preparation compared to traditional materials like amorphous selenium and Cd(Zn)Te. However, challenges related to device operation, excessively high dark currents, and long‐term stability have delayed commercialization. Here, two novel semiconductors incorporating stable sulfonium cations are presented, [(CH_3_CH_2_)_3_S]_6_Bi_8_I_30_ and [(CH_3_CH_2_)_3_S]AgBiI_5_, synthesized via solvent‐free ball milling and fabricated into dense polycrystalline pellets using cold isostatic compression, two techniques that can easily be upscaled, for X‐ray detection application. The fabricated detectors exhibit exceptional sensitivities (14 100–15 190 µC Gy_air_^−1^ cm^−2^) and low detection limits (90 nGy_air_ s^−1^ for [(CH_3_CH_2_)_3_S]_6_Bi_8_I_30_ and 78 nGy_air_ s^−1^ for [(CH_3_CH_2_)_3_S]AgBiI_5_), far surpassing current commercial detectors. Notably, they maintain performance after 9 months of ambient storage. The findings highlight [(CH_3_CH_2_)_3_S]_6_Bi_8_I_30_ and [(CH_3_CH_2_)_3_S]AgBiI_5_ as scalable, cost‐effective and highly stable alternatives to traditional semiconductor materials, offering great potential as X‐ray detectors in medical and security applications.

## Graphical abstract

[(CH_3_CH_2_)_3_S]_6_Bi_8_I_30_ and [[(CH_3_CH_2_)_3_S]AgBiI_5_ are introduced as novel X‐ray detector materials with exceptional sensitivity and low detection limits. Produced via scalable, green synthesis, they remain highly durable after 9 months of storage and continuous X‐ray use, setting a new benchmark for medical imaging and outperforming many existing and emerging technologies.

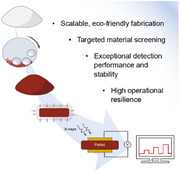

## 1 Introduction

Semiconductor X‐ and gamma‐ray (X/γ‐ray) detectors with high performance and durability are critical across numerous fields, including medical imaging, non‐destructive testing, security, nuclear industries, and scientific research. Traditional materials such as amorphous selenium (a‐Se) and Cd(Zn)Te have been widely used for direct X/γ‐ray conversion,^[^ ^1^ ^]^ but issues persist regarding their complex crystal growth processes and operational requirements, for instance strong electric fields. In medical imaging, in particular, the need for detectors with higher sensitivity and lower detection limits is highly desirable, as these would enable the use of lower radiation doses, thereby reducing patient exposure. Key performance indicators (KPIs) for X‐ray detectors include sensitivity, the limit of detection (LoD), mobility‐lifetime product (*µτ*), and resistivity. Sensitivity measures the detector's ability to convert X‐ray photons into an electric signal, while the LoD indicates the minimum detectable radiation above the background noise. Since 2013, there has been a revival in the field of new X‐ray detector materials after lead‐based metal halide perovskites were reported as promising alternatives for X‐ray detection due to their high resistivity, excellent *µτ* products, high atomic numbers (*Z*), and ease of synthesis.^[^ ^2^, ^3^ ^]^ However, their instability under ambient conditions and the high dark currents remains a significant limitation for commercialization.^[^ ^4^ ^]^

Bismuth‐based (Bi‐based) organic‐inorganic hybrid materials have emerged as a promising alternative, offering superior thermal and moisture stability compared to lead‐based perovskites.^[^ ^5^ ^]^ These materials are often synthesized via low‐temperature, non‐vacuum methods, making them cost‐effective alternatives.^[^ ^5^, ^6^, ^7^, ^8^ ^]^ Bi‐halides, in particular, display favorable bandgaps (1.8–2.5 eV) and high atomic numbers (*Z* = 83 for Bi, *Z* = 53 for I), contributing to low thermal noise and high X‐ray absorption. These properties make them ideal for X‐ray detection applications, where high‐*Z* materials are preferred for their superior stopping power and sensitivity.^[^ ^9^ ^]^ Among the most studied Bi‐halides is Cs_2_AgBiBr_6_, a double perovskite known for its indirect bandgap, long charge‐carrier lifetimes, and excellent stability. Cs_2_AgBiBr_6_ has been evaluated as an X‐ray detector in the form of single crystals,^[^ ^8^ ^]^ films,^[^ ^10^ ^]^ and thick pellets,^[^ ^7^ ^]^ showing sensitivities of up to 1 974 µC Gy_air_^−1^ cm^−2^ in single‐crystal detectors.^[^ ^11^ ^]^ Furthermore, other Bi‐based materials, such as MA_3_Bi_2_I_9_, BiOI, Cs_3_Bi_2_I_9_, and Rb_3_Bi_2_I_9_, have shown promising properties, with reported sensitivities as high as 10 620 µC Gy_air_^−1^ cm^−2^ for MA_3_Bi_2_I_9_ in single crystal form.^[^ ^12^, ^13^, ^14^ ^]^ Single crystals (SCs) generally outperform other forms, given their superior crystal quality, absence of grain boundaries, and minimal defects.^[^ ^15^ ^]^ However, scaling SCs for practical applications remains challenging due to the difficulty of growing large crystals using conventional techniques like the Bridgman and Czochralski methods, which are both time‐consuming and costly, limiting their scalability for practical use.^[^ ^16^ ^]^

**Figure 1:**
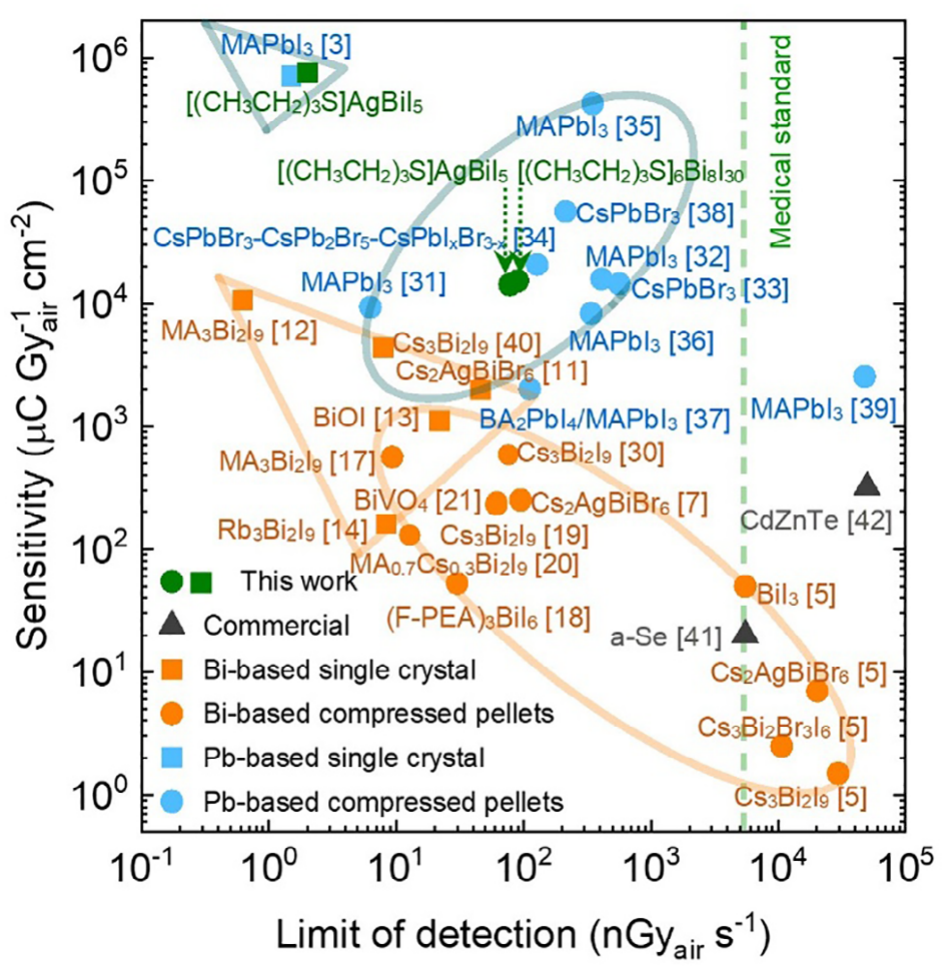
X‐ray detector performance of organic‐inorganic hybrid materials based on Bi‐based^[^ ^5^, ^7^, ^17^, ^18^, ^19^, ^20^, ^21^, ^30^ ^]^ and Pb‐based^[^ ^31^, ^32^, ^33^, ^34^, ^35^, ^36^, ^37^, ^38^, ^39^ ^]^ compressed pellets (CP), solution‐grown Bi‐based^[^ ^11^, ^12^, ^13^, ^14^, ^40^ ^]^ and Pb‐based^[^ ^3^ ^]^ single crystals (SC), commercially available amorphous selenium (a‐Se)^[^ ^41^ ^]^ and polycrystalline Cd(Zn)Te^[^ ^42^ ^]^ with sensitivity and detection limits reported in the literature, as compared to the results obtained in this work for [(CH_3_CH_2_)_3_S]_6_Bi_8_I_30_ and [(CH_3_CH_2_)_3_S]AgBiI_5_ compressed pellets, as well as on [(CH_3_CH_2_)_3_S]AgBiI_5_ as single crystal. Bi‐based compressed pellets (highlighted by orange oval) exhibit sensitivity and detection limits comparable to single‐crystal detectors (highlighted by orange triangle). However, Bi‐based detectors remain inferior to the Pb‐based counterparts (orange and blue symbols). Notably, the hybrid Bi‐based detectors presented in this study are comparable in performance to Pb‐based detectors reported in the literature.

In contrast, polycrystalline pellets present a practical alternative for flat panel X‐ray detectors, as they can be fabricated quickly and cost‐effectively using hydraulic compression, which enables the production of thick samples essential for effective X‐ray absorption. Although pellets typically exhibit lower performance than SCs due to the presence of grain boundaries (which is generally absent in SCs), known to be sites of recombination losses, recent studies have demonstrated that Bi‐based materials in pellet form still show promising sensitivities and LoDs, often outperforming traditional materials such as a‐Se and Cd(Zn)Te (**Figure** **1**; Table S1, Supporting Information).^[^ ^5^, ^7^, ^17^, ^18^, ^19^, ^20^, ^21^ ^]^ Moreover, lead‐based perovskites, such as MAPbI_3_, have shown comparable results in pellet form, further encouraging research in this area. The trend of SC detectors displaying better performance than the same material in compressed pellet form can be observed in Figure 1 when comparing the sensitivities and detection limits of the materials studied in the form of SCs and compressed pellets. Despite the advancements in Bi‐based materials, there is still a need to explore new compositions and structures to expand the portfolio of potential X‐ray detector materials. Bi‐halides, with their diverse structural motifs—from 0D cluster anions to 2D frameworks—offer rich opportunities for tuning material properties and optimizing the performance for specific applications.^[^ ^22^ ^]^ Furthermore, the role of the organic cations in shaping the inorganic structural framework and impacting optoelectronic performance is not yet fully understood, especially for sulfonium cations, which have been largely overlooked in perovskite‐ and Bi‐based hybrids. Recent results suggest that, due to their superior chemical resistance and ambient stability, sulfonium cations could be highly effective in enhancing long‐term device stability.^[^ ^23^, ^24^ ^]^ Unlike the more conventional protic ammonium cations, sulfonium cations are aprotic and chemically resistant to moisture‐induced degradation due to the absence of hydrogen bonding.^[^ ^25^, ^26^ ^]^ Moreover, the sulfur atoms form strong electrostatic interactions directly with the halides of the inorganic framework, leading to compact anionic motifs with short interlayer distances, whereas ammonium cations interact through hydrogen bonding, resulting in distinctly different structures. Sulfonium‐based hybrid materials have demonstrated intriguing properties, including broad red emission in a lead‐based perovskite‐like material and photochromism in polyoxometalates through electron transfer involving sulfonium cations.^[^ ^27^, ^28^ ^]^ These attributes render sulfonium‐based hybrid materials promising candidates for exploration in advanced applications. Understanding and deriving structure‐property relationships in these materials will be key to unlocking their full potential. By correlating the unique structural features of Bi‐halides with their electronic, optic, and transport properties, future studies can guide the rational design of materials that meet the specific requirements of high‐performance X‐ray detectors. This approach will facilitate the development of tailored materials with enhanced sensitivity, stability, and operational efficiency.

**Figure 2:**
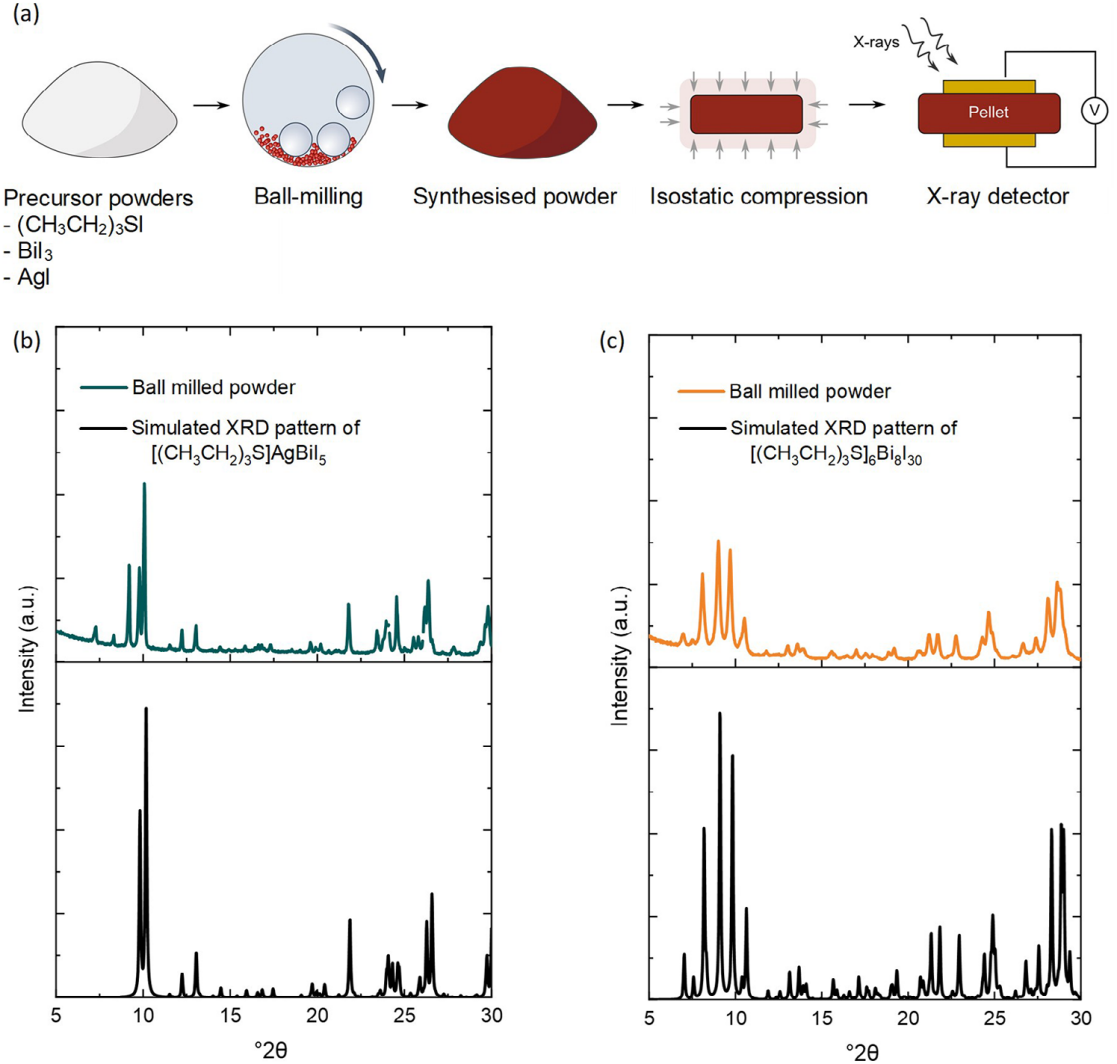
Fabrication and Characterization of [(CH_3_CH_2_)_3_S]_6_Bi_8_I_30_ and [(CH_3_CH_2_)_3_S]AgBiI_5_. a) Schematic illustration of the device fabrication process, where powders of the materials were first synthesized by ball‐milling and subsequently subjected to a pressure of ≈1 GPa using a hydraulic press, followed by the deposition of gold contacts to fabricate X‐ray detector devices. b) and c) Experimental and simulated PXRD pattern of [(CH_3_CH_2_)_3_S]AgBiI_5_ and [(CH_3_CH_2_)_3_S]_6_Bi_8_I_30_.

In this study, we introduce two novel hybrid semiconductors, [(CH_3_CH_2_)_3_S]_6_Bi_8_I_30_ and [(CH_3_CH_2_)_3_S]AgBiI_5_, as promising candidates for X‐ray detection. These materials were identified during a broader exploration of perovskite‐inspired compounds for energy applications.^[^ ^29^ ^]^ The high atomic numbers of the constituent atoms, suitable bandgaps, and unique structural features—incorporating stable sulfonium cations over traditionally used hygroscopic ammonium cations—make them ideal for X‐ray detection. Structurally, [(CH_3_CH_2_)_3_S]_6_Bi_8_I_30_ comprises 0D polynuclear iodobismuthates, while [(CH_3_CH_2_)_3_S]AgBiI_5_ contains 2D bimetallic Ag/Bi‐iodides. Both materials were synthesized via a solvent‐free mechanochemical method, producing polycrystalline powders that were subsequently compressed into dense pellets using isostatic compression (**Figure** **2**a). This combined process is not only scalable but also well‐established in industry, ensuring precise stoichiometric control and rapid production for large‐scale applications. The combination of mechanosynthesis and isostatic compression provides an efficient and scalable approach for screening and evaluating new materials for X‐ray detectors—a method underexplored in the literature. Furthermore, SCs of [(CH_3_CH_2_)_3_S]AgBiI_5_ were grown and evaluated for X‐ray detection to enable a thorough performance comparison with the compressed pellet version. The resulting detectors, assembled with symmetric gold contacts, demonstrate high resistivity, excellent *µτ* products, and superior sensitivity compared to commercially used materials, such as a‐Se and Cd(Zn)Te, as well as MAPbI_3_ in compressed pellet form. In addition, the materials exhibit remarkable stability after 9 months of ambient storage, highlighting their potential for commercial X‐ray detection technologies. These results underscore the potential of these materials, achieved without post‐synthesis treatments, and the methodologies used for future application in X‐ray detectors for medical imaging and other fields.

## 2 Results and Discussion

### 2.1 Material Synthesis, Pellet Fabrication and Optoelectronic Properties

Single crystals of [(CH_3_CH_2_)_3_S]_6_Bi_8_I_30_ and [(CH_3_CH_2_)_3_S]AgBiI_5_ were synthesized using a wet‐chemical method, and their structures were thoroughly characterised in previous studies.^[^ ^29^ ^]^ However, achieving pure phases during wet‐chemical synthesis often poses challenges, frequently leading to the formation of unknown competing phases. To overcome this, we here introduced a dry mechanochemical ball‐milling method as an alternative. This solid‐state technique offers precise stoichiometric control and scalability, making it more suitable for commercialisation compared to solution‐based methods.^[^ ^43^ ^]^ Furthermore, it is highly suitable for synthesising complex materials, including metastable phases, as it allows stoichiometric control of the precursors and control over the reaction conditions, such as the frequency and reaction time. The potential to analyse the reaction outcomes at specific time intervals renders it highly appealing for controlled product formation. Mechanochemical synthesis also provides an environmentally friendly approach, facilitating swift industrial adoption. In our experiments, precursors were mixed in stoichiometric amounts and subjected to short milling times. Early trials using 30 min of milling were noted to be insufficient for complete reactions, resulting in the formation of impurities. However, extending the reaction time to 1 h resulted in the formation of the desired target compounds, which were confirmed via powder X‐ray diffraction (PXRD) analysis (Figure 2b,c). The PXRD patterns matched the reference patterns obtained from the structural refinement of single‐crystal X‐ray data.^[^ ^29^ ^]^ [(CH_3_CH_2_)_3_S]_6_Bi_8_I_30_ came out phase pure, while minor crystalline impurities were detected in [(CH_3_CH_2_)_3_S]AgBiI_5_. One impurity, corresponding to the two peaks at around 2θ = 7.25 and 9.3, was identified as [(CH_3_CH_2_)_3_S]Ag_4_I_5_, previously reported by Kloo et al.^[^ ^44^ ^]^ The resulting powders were compressed into pellets at room temperature and ≈1 GPa of pressure, with thicknesses between 1–2 mm, for use in X‐ray detector devices. Figure 2a graphically illustrates the fabrication process of the X‐ray detector devices. In parallel, SCs of [(CH_3_CH_2_)_3_S]AgBiI_5_ were grown according to our previously reported method to enable a comparison and to better understand its potential as an X‐ray detector material.^[^ ^29^ ^]^ Bandgap estimation via diffuse reflectance spectroscopy combined with the Kubelka‐Munk function^[^ ^45^ ^]^ yielded bandgaps of 1.80 eV for [(CH_3_CH_2_)_3_S]AgBiI_5_ and 1.98 eV for [(CH_3_CH_2_)_3_S]_6_Bi_8_I_30_ (Figure S1, Supporting Information), which are optimal for X‐ray detection applications. These values suggest effective X‐ray photon absorption and electron‐hole pair generation, while minimizing thermal noise. The subsequent sections will detail the evaluation of these materials’ performance as X‐ray detectors.

### 2.2 Resistivity and *µτ* Product

**Figure 3:**
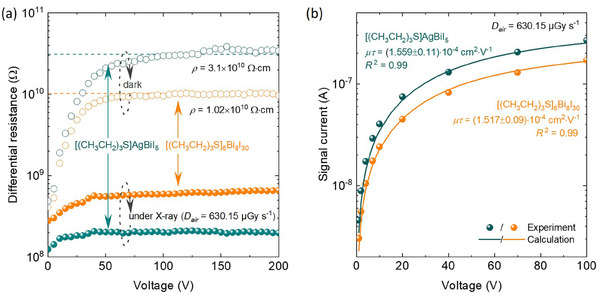
Electrical and photoelectric properties of the Au/[(CH_3_CH_2_)_3_S]_6_Bi_8_I_30_/Au and Au/[(CH_3_CH_2_)_3_S]AgBiI_5_/Au X‐ray detector devices. a) Typical voltage dependence of the differential resistance of the Au/[(CH_3_CH_2_)_3_S]_6_Bi_8_I_30_/Au and Au/[(CH_3_CH_2_)_3_S]AgBiI_5_/Au detectors in the dark (blank circles) and under X‐rays at a dose rate of 630.15 µGy_air_ s^−1^ (filled circles); b) Typical bias voltage‐dependent signal current plots of the Au/[(CH_3_CH_2_)_3_S]_6_Bi_8_I_30_/Au and Au/[(CH_3_CH_2_)_3_S]AgBiI_5_/Au X‐ray detectors (circles) and calculated by the Hecht equation and the derived µ*τ* product.

The first two key performance indicators (KPIs) used to assess the X‐ray detection capabilities of the device are (i) resistivity (*ρ*), which contributes to low dark currents, and (ii) the *µτ* product, which reflects charge collection efficiency, which can be estimated using the Hecht equation.^[^ ^33^ ^]^ Resistivity is estimated from *ρ* = *R_diff_ ⋅ A/d*, where *R_diff_* is the differential resistance, *A* is the metal contact area, and *d* is the thickness. A high resistivity and a large *µτ* product indicate the potential for high sensitivity, a large signal‐to‐noise ratio (SNR), and a low limit of detection (LoD). The differential resistance, *R_diff_ = *∂*V*/∂*I*, was extracted from the current‐voltage (*I‐V*) characteristics (**Figure** **3**a; Note S1, Supporting Information). The constant *R_diff_* observed in the voltage range of 0–200 V confirms the high quality of the Au ohmic contacts and the absence of minority charge‐carrier injection.^[^ ^46^, ^47^ ^]^

The resistivity of [(CH_3_CH_2_)_3_S]_6_Bi_8_I_30_ and [(CH_3_CH_2_)_3_S]AgBiI_5_ compressed pellet detectors varies over a range of (0.25–5.66) × 10¹⁰ Ω cm and (1.27–5.32) × 10¹⁰ Ω cm, with mean values of (1.18 ± 0.76) × 10¹⁰ Ω cm (*n* = 7) and (3.1 ± 0.60) × 10¹⁰ Ω cm (*n* = 6), respectively (Figure S2c,d, Supporting Information). These values are comparable to or exceed previously reported values for sintered compressed pellets.^[^ ^7^, ^19^, ^30^, ^31^, ^32^, ^33^, ^34^ ^]^ Surprisingly, the resistivity of [(CH_3_CH_2_)_3_S]AgBiI_5_ is approximately 2.5 times higher than that of [(CH_3_CH_2_)_3_S]_6_Bi_8_I_30_ (Table S1; Figure S2c, Supporting Information). While the larger bandgap of [(CH_3_CH_2_)_3_S]_6_Bi_8_I_30_ would predict higher resistivity, the polycrystalline nature of the pellets results in significant defect concentrations, which dominate the electrical conductivity. Therefore, the variation in resistivity is primarily attributed to differences in defect concentrations rather than bandgap differences.

The µ*τ* product represents an estimate of the transport properties of charge carriers generated by X‐ray illumination, reflecting the probability of electrons and holes reaching the electrodes before recombination. Figure 3b shows the calculated µ*τ* products using a simplified Hecht equation, which models a single type of charge carrier in a detector with two ohmic contacts and a uniform electric field.^[^ ^40^, ^48^, ^49^, ^50^ ^]^ The µ*τ* products, estimated to be (1.52 ± 0.09) × 10⁻⁴ cm^2^ V⁻¹ for [(CH_3_CH_2_)_3_S]_6_Bi_8_I_30_ and (1.56 ± 0.11) × 10⁻⁴ cm^2^ V⁻¹ for [(CH_3_CH_2_)_3_S]AgBiI_5_, indicate efficient charge transport and collection. These values compare favorably with or exceed record values for Bi‐based pellets that were fabricated using extensive post‐treatment methods (Table S1, Supporting Information),^[^ ^17^, ^19^, ^21^ ^]^ and are significantly higher than for a‐Se (10⁻⁷ cm^2^ V⁻¹).^[^ ^41^ ^]^ Notably, the µ*τ* product for the [(CH_3_CH_2_)_3_S]AgBiI_5_ SC is about 1.5 times higher than the compressed pellet equivalents, indicating more efficient charge transport and improved sensitivity in SCs.

Approximately 63% of 8.05 keV (Cu Kα) photons are absorbed within the first ≈13 µm of the detector, corresponding to ≈1% of a 1 mm thick compressed pellet (see Supplementary Note 2). As a result, charge carriers must drift across nearly the entire pellet thickness to reach the back electrode. Increasing the photon energy to 35 keV (corresponding to the Bremsstrahlung background peak under a 40 kV X‐ray tube voltage) and further to 100 keV extends the attenuation depth to ≈0.11 and ≈1.3 mm, respectively (Figure S3a,b, Supporting Information), is expected to improve charge collection, even at low bias voltages. Hence, the obtained µ*τ* product values indicate excellent charge transport properties already under relatively low‐energy X‐ray photon irradiation.

With these known µ*τ* products, the statistical distance that photogenerated charge carriers can travel before recombination, known as the Schubweg, can be estimated.^[^ ^5^, ^51^ ^]^ For an applied electric field of 500–1000 V cm⁻¹, the Schubweg is estimated to be 0.8‐1.5 mm, which is nearly equal to the sample thickness, ensuring efficient charge collection across the detector.

### 2.3 Sensitivity and LoD

**Figure 4:**
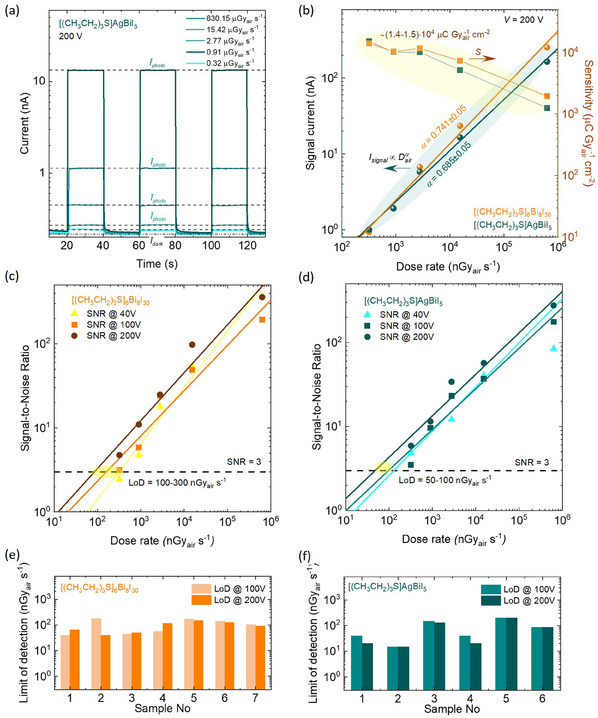
Performance of [(CH_3_CH_2_)_3_S]_6_Bi_8_I_30_ and [(CH_3_CH_2_)_3_S]AgBiI_5_ as X‐ray detectors. a) X‐ray‐induced response of the Au/[(CH_3_CH_2_)_3_S]AgBiI_5_/Au detectors at different dose rates at a given bias voltage of 200 V. Dashed lines indicate the mean values of photocurrent and dark current. b) Dose rate‐dependent signal current (circles) and sensitivity (squares) of the X‐ray detectors based on [(CH_3_CH_2_)_3_S]AgBiI_5_ (green symbols) and [(CH_3_CH_2_)_3_S]_6_Bi_8_I_30_ (orange symbols) at a bias voltage of 200 V. Approximations of the *I_signal_ *∝ (*D_air_*)*^α^* dependencies are shown by solid lines. c) and d) X‐ray dose rate dependent signal‐to‐noise ratio of the detectors under the bias voltage from 1 to 200 V. The dashed line represents an SNR of 3, and thus the detection limit is in the range of (100–300) nGy_air_ s^−1^ and (50‐100) nGy_air_ s^−1^ for the [(CH_3_CH_2_)_3_S]_6_Bi_8_I_30_‐based and [(CH_3_CH_2_)_3_S]AgBiI_5_‐based detectors, respectively. e) and f) distribution of the limit of detection of two sets of [(CH_3_CH_2_)_3_S]_6_Bi_8_I_30_ (e) and [(CH_3_CH_2_)_3_S]AgBiI_5_ (f) Bar charts of the detection limit of compressed pellets under bias voltages of 100 V and 200 V.

In addition to the charge‐transport properties discussed earlier, sensitivity and LoD are critical KPIs for X‐ray detectors. We evaluated the sensitivity of the [(CH_3_CH_2_)_3_S]_6_Bi_8_I_30_‐ and [(CH_3_CH_2_)_3_S]AgBiI_5_‐based detectors to Cu anode radiation (primary Kα (8.05 keV) emission line) and to synchrotron radiation, using photon energies of 8 and 12 keV (see Supplementary Note 3). In practise, it is important that these detectors can detect X‐rays at the lowest possible dose rates that the used experimental setups can generate, which is ≈20 times lower than the typical medical diagnostic rate of 5.5 µGy_air_ s⁻¹ (dashed line in Figure 1), helping to minimise radiation exposure.^[^ ^52^ ^]^ The X‐ray photocurrent showed a good on‐off response, increasing with both dose rate (**Figure** **4**a,b, and **5**b **inset**) and applied electric field (Figure S6a, Supporting Information). The current response time evaluation indicates that the [(CH_3_CH_2_)_3_S]AgBiI_5_ device has an on/off response time of 41.0 ms/47.3 ms, while the [(CH_3_CH_2_)_3_S]_6_Bi_8_I_30_ device shows an on/off response time of 27.3 ms/28.4 ms (Figure S6e,f, Supporting Information). Notably, these response times are faster than those reported for the well‐known CsPbBr_3_,^[^ ^38^ ^]^ demonstrating our detectors' competitive performance and suitability for applications requiring rapid and efficient X‐ray signal detection.

**Figure 5:**
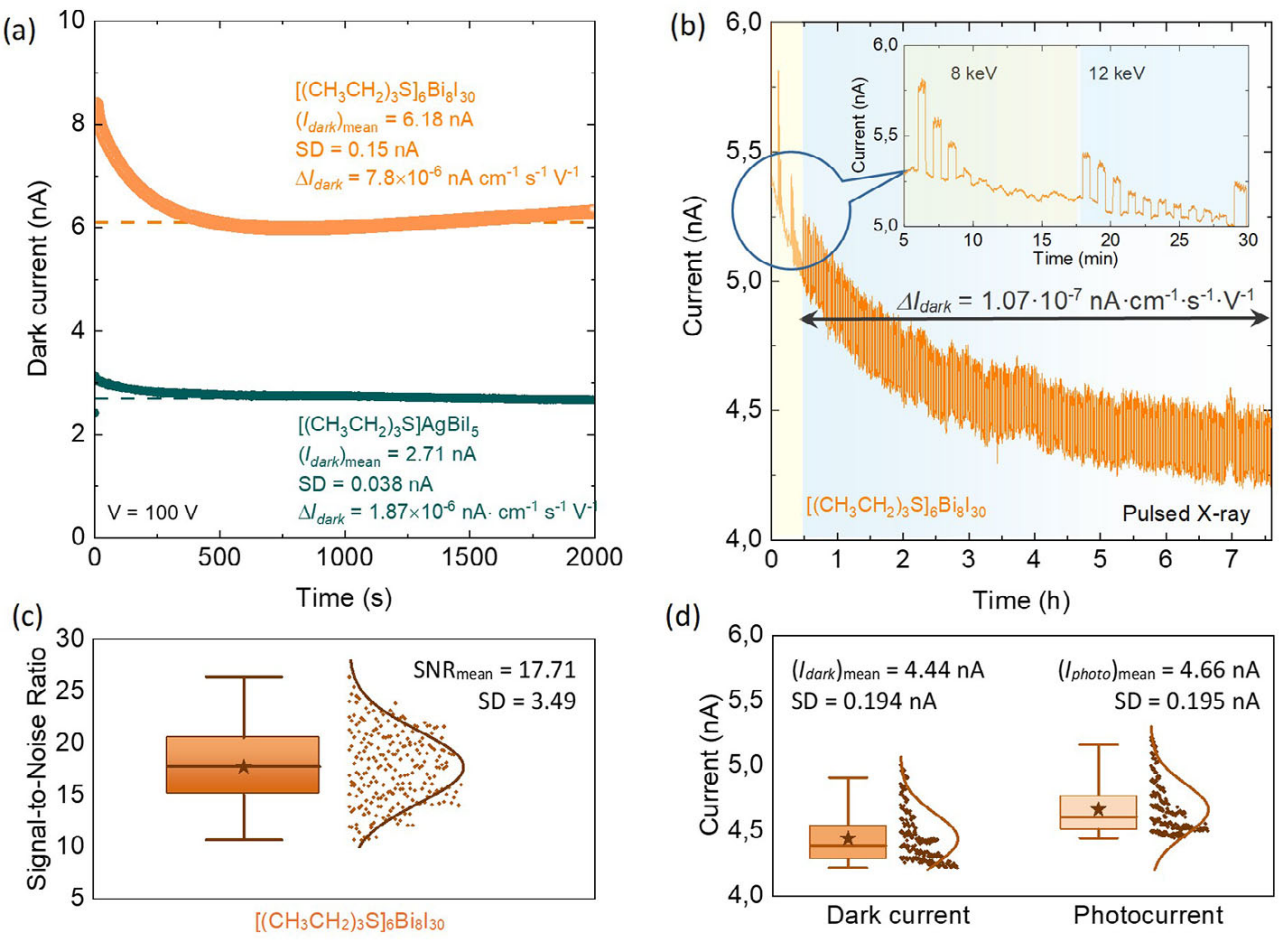
Irradiation‐ and bias stability of the [(CH_3_CH_2_)_3_S]_6_Bi_8_I_30_‐ and [(CH_3_CH_2_)_3_S]AgBiI_5_‐based detectors. a) Typical curves of dark current drift of the Au/[(CH_3_CH_2_)_3_S]_6_Bi_8_I_30_/Au and Au/[(CH_3_CH_2_)_3_S]AgBiI_5_/Au X‐ray detectors under a bias voltage of 100 V during a measurement period of 2000 s. The dark current drift (*ΔI*), mean stabilized dark current (*I_dark_*) and its standard deviation (SD) are also provided; b) X‐ray photoresponse characteristics of the Au/[(CH_3_CH_2_)_3_S]_6_Bi_8_I_30_/Au X‐ray detector under 200 V bias voltage during 7.5 h of 40 s on/off cycles. The inset illustrates the first 30 min of the experiment, where the photon intensity decreased in a 40 s on/off cycle, initially at a photon energy of 8 keV and later at 12 keV. For the remaining 7 h, the detector was exposed to unattenuated 12 keV photons in the same cycle; c) and d) – SNR (c), and dark current and photocurrent (d) fluctuation during 7 h of measurement under synchrotron irradiation.

The signal current (*I_signal_*) was calculated by subtracting the average dark current (*Ī_dark_*) from the average photocurrent (*Ī_photo_*) (Figure 4a; Figure S6b, Supporting Information). The relationship between *I_signal_* and dose rate follows a sublinear power law, *I_signal_* ∝ *D_air_^α^*, with *α* values of 0.741 ± 0.05 for Au/[(CH_3_CH_2_)_3_S]_6_Bi_8_I_30_/Au and 0.685 ± 0.05 for Au/[(CH_3_CH_2_)_3_S]AgBiI_5_/Au, respectively, likely indicating carrier trapping (Figure 4b; Figure S6c, Supporting Information).

The X‐ray sensitivity, *S*, is defined as the current density per unit radiation exposure^[^ ^39^, ^41^, ^46^, ^47^, ^48^ ^]^ (see Methods for details) and was evaluated for the compressed pellets of both materials and also for SCs of [(CH_3_CH_2_)_3_S]AgBiI_5_. As expected from the µ*τ* evaluation in the previous section, the SCs display significantly higher sensitivity, ≈50 times greater than the compressed pellets, which correlates well with the observed trend illustrated in Figure 1. The sensitivity reached ≈7.5⋅10^5^ µC Gy_air_^−1^ cm^−2^ for the [(CH_3_CH_2_)_3_S]AgBiI_5_ single crystal, as compared to ≈(1.4–1.5)⋅10^4^ µC Gy_air_^−1^ cm^−2^ for the compressed pellets at low radiation intensities (Figure 4b; Figure S6d, Supporting Information). To the best of our knowledge, the latter sensitivities are the highest reported thus far for Bi‐based compressed pellet materials (Figure 1; Table S1, Supporting Information). Despite the significantly better SC performance, the more straightforward and more accessible fabrication of compressed pellets offsets the sensitivity loss, as they still provide a sensitivity of ≈10^4^ µC Gy_air_^−1^ cm^−2^, independent of dose rate within the range used for medical imaging (<5.5 µGy_air_ s^−1^)^[^ ^52^ ^]^ (Figure 4b). This independence simplifies operation and eliminates the need for complex calibration of signal output to dose rates, enhancing their potential for medical applications.

In summary, the [(CH_3_CH_2_)_3_S]_6_Bi_8_I_30_‐ and [(CH_3_CH_2_)_3_S]AgBiI_5_‐based devices exhibit remarkable sensitivity, surpassing other Bi‐based materials, such as Cs_3_Bi_2_I_9_, Cs_2_AgBiBr_6_, BiVO_4_, and most lead‐based perovskites (e.g., MAPbI_3_ in pellet form) (Figure 1; Table S1, Supporting Information). At 200 V and a dose rate of 321 nGy_air_ s^−1^, the sensitivity is ≈40 times higher than for polycrystalline Cd(Zn)Te (318 µC Gy_air_^−1^ cm^−2^)^[^ ^42^ ^]^ and ≈700 times higher than for a‐Se detectors (20 µC Gy_air_^−1^ cm^−2^), which require much stronger electric fields (10^5^ V cm⁻¹).^[^ ^41^ ^]^ This can be explained by the presence of photoconductive gain in the devices along with a µ*τ* product several orders of magnitude higher than that of a‐Se. We speculate that charge‐carrier traps within the detector materials, with concentrations of 1.15 × 10¹¹ cm⁻^3^ for [(CH_3_CH_2_)_3_S]AgBiI_5_ and 9.82 × 10¹⁰ cm⁻^3^ for [(CH_3_CH_2_)_3_S]_6_Bi_8_I_30_ (Figure S8, Supporting Information), are responsible for inducing photoconductive gain under high bias voltage (see Methods and Supplementary Note 4 for details).^[^ ^5^, ^9^, ^38^, ^53^, ^54^, ^55^ ^]^

The LoD, another crucial KPI, particularly for medical imaging and security screening, was determined as the dose rate at which the signal‐to‐noise ratio (SNR) equals 3 (Figure 4c,d; Figure S7, Supporting Information). The mean LoD values were 90 nGy_air_ s^−1^ for [(CH_3_CH_2_)_3_S]_6_Bi_8_I_30_ and 78 nGy_air_ s^−1^ for [(CH_3_CH_2_)_3_S]AgBiI₅ (compressed pellets), slightly higher than for [(CH_3_CH_2_)_3_S]AgBiI₅ single crystals (≈10–20 nGy_air_ s^−1^), but comparable to other Bi‐based detectors based on compressed pellets (Figure 1; Table S1, Supporting Information). These values are 2–4 times lower than those for Pb‐based perovskite pellets and 55–500 times lower than for a‐Se and Cd(Zn)Te detectors, respectively (Figure 1; Table S1, Supporting Information).

The estimated LoD of these detectors is approximately 60–70 times lower than the threshold required for medical diagnostics, enabling reduced radiation dosage in routine X‐ray examination, thereby lowering the risk of radiation‐induced cancer.^[^ ^52^ ^]^ Increasing the electric field does not significantly improve LoD (Figure 4c,d), but it does enhance sensitivity, reaching 14 100–15 190 µC Gy_air_^−1^ cm^−2^ at a dose rate of 321 nGy_air_ s^−1^ (Figure 4b). Even at low bias voltages (e.g. 40 V), the LoD remains favorable (Figure 4c,d), with the sensitivity only reduced fourfold, maintaining a high range of 3 000‐5 000 µC Gy_air_^−1^ cm^−2^ (Figure S6d, Supporting Information). This robust sensitivity at reduced electric fields highlights the suitability of the detectors for low‐dose X‐ray applications without compromising detection efficiency.

The exceptional sensitivity and low detection limits observed, even at moderate X‐ray energies, underscore the potential of these materials for diverse applications, including security screening, elemental analysis, and particularly medical imaging. Their performance advantage likely arises from the unique structural features, such as the short interlayer distances between the inorganic components,^[^ ^29^ ^]^ which have been shown to be crucial in enhancing X‐ray detection capabilities.^[^ ^56^, ^57^ ^]^

### 2.4 Stability

The environmental stability of perovskite‐based devices is a well‐recognized challenge due to performance degradation during ageing. However, Bi‐based detectors have shown promising long‐term stability and self‐healing properties, which we further explored in our Au/[(CH_3_CH_2_)_3_S]_6_Bi_8_I_30_/Au and Au/[(CH_3_CH_2_)_3_S]AgBiI_5_/Au devices. These detectors were stored for 9 months under ambient conditions (≈21 °C, 70% relative humidity) without protective encapsulation. Remarkably, their performance remained stable, with only minimal changes in sensitivity and detection limit over time (Figure S8, Supporting Information).

The detectors exhibit a good dark current stability with an initial exponential decrease in dark current under a bias voltage of 100 V, followed by stabilisation after ≈5 min, reaching equilibrium within 20–30 min. The stabilized dark current averaged 2.71 and 6.18 nA for Au/[(CH_3_CH_2_)_3_S]AgBiI_5_/Au and Au/[(CH_3_CH_2_)_3_S]_6_Bi_8_I_30_/Au, respectively. Current drift values of Δ*I* = 7.8 × 10⁻⁶ nA cm⁻¹ s⁻¹ V⁻¹ for Au/[(CH_3_CH_2_)_3_S]_6_Bi_8_I_30_/Au and Δ*I *= 1.87 × 10⁻⁶ nA cm⁻¹ s⁻¹ V⁻¹ for Au/[(CH_3_CH_2_)_3_S]AgBiI_5_/Au were obtained (**Figure** **5**a, see Experimental Section for details), which are practically negligible and within the same range as post‐treated devices, such as BiOBr‐passivated Cs₂AgBiBr₆ and BiVO₄ compressed pellets.^[^ ^7^, ^21^ ^]^ The Au/[(CH_3_CH_2_)_3_S]AgBiI_5_/Au and Au/[(CH_3_CH_2_)_3_S]_6_Bi_8_I_30_/Au detectors also display good stability under steady X‐ray illumination. As seen in Figure S2e (Supporting Information), under 200 V bias and 3059.418 µGy_air_ s^−1^ cm^−2^ X‐ray illumination, the devices can deliver a stable photocurrent for 2000 s (total X‐ray dose of 1260 mGy_air_), which is equal to the dose of 340 two‐view digital mammography examinations.^[^ ^58^ ^]^

Under long‐term synchrotron irradiation, the detectors also demonstrated stable performance with high signal‐to‐noise ratios and minimal baseline drift over a 7‐h period (total absorbed dose of **234.36 Gy_air_**) (Figure 5b). Specifically, the Au/[(CH_3_CH_2_)_3_S]_6_Bi_8_I_30_/Au detector displayed a baseline drift of 1.07 × 10⁻⁷ nA cm⁻¹ s⁻¹ V⁻¹, ≈8 times lower than BiOBr‐passivated Cs₂AgBiBr₆ devices. All detectors maintained a consistent SNR throughout the testing period, averaging to 17.7 (Figure 5c), and a stable photoresponse, reinforcing the robustness of the detectors even 9 months after fabrication (Figure S12, Supporting Information). The fluctuations in the sensitivity and the LoD values observed in Figure S12 (Supporting Information) are primarily attributed to instrumental variations and differences in experimental conditions during the repeated measurements over the 9 months.

The impressive stability of these materials likely stems from their structural features, with sulfonium cations providing enhanced stability over the ammonium counterparts by minimising hydrogen bonding and thus improving moisture stability. In addition, short interlayer distances between the inorganic components^[^ ^29^ ^]^ likely contribute further to the robustness of the materials.

## 3 Conclusion

This study presents two new bismuth‐based compounds, [(CH_3_CH_2_)_3_S]_6_Bi_8_I_30_ and [(CH_3_CH_2_)_3_S]AgBiI_5_, synthesized via an efficient mechanochemical ball‐milling process and fabricated as polycrystalline pellets for X‐ray detection. These materials, prepared without post‐treatment, exhibit excellent charge‐transport properties (µ*τ* products of 1.52 × 10⁻⁴ and 1.56 × 10⁻⁴ cm^2^ V⁻¹), remarkably high sensitivity (14 100–15 190 µC Gy_air_⁻¹ cm^−^^2^), and low detection limits (90 and 78 nGy_air_ s⁻¹), outperforming commercial a‐Se‐based detectors. The stability of these new detectors over 9 months in ambient storage, combined with the negligible current drifts under constant irradiation and bias voltage, underscores their robustness for scalable, long‐term use in X‐ray detection, particularly in medical imaging.

Beyond immediate applications, this work sheds light on sulfonium‐based hybrid materials, suggesting new guidelines for designing materials with targeted properties across multiple energy application areas. Mechanochemical synthesis combined with isostatic compression also emerges as an effective screening tool for new materials of specific compositions for subsequent bulk material property Characterization, not only for X‐ray detection but also for various optoelectronic applications. Future studies will focus on optimising device architecture, including the use of asymmetric contacts and passivation layers, and exploring the materials’ performance under high‐energy radiation.

## 4 Experimental Section

### Chemicals

Triethylsulfonium iodide ((CH_3_CH_2_)_3_SI, 97%, Thermo Scientific), Silver iodide (AgI, 99%, Sigma Aldrich), and bismuth iodide (BiI_3_, 98%, TCI) were used as received without any further purification.

### Mechanosynthesis and Hydraulic Compression

The precursor salts were mixed in stoichiometric amounts inside 10 ml stainless steel vessels for solvent‐free mechanosynthesis. The reactions were performed by neat grinding in a vibration ball mill (Pulverisette 23, Fritsch) at 50 Hz for 1 h under ambient conditions to generate polycrystalline powders of [(CH_3_CH_2_)_3_S]_6_Bi_8_I_30_ and [(CH_3_CH_2_)_3_S]AgBiI_5_. To form compressed pellets of each material, the powders were placed in a 10 mm die set and pressed in a hydraulic press (MP150D, Maassen) at 8 tons for 30 s.

### Single Crystal Growth

Single crystals of [(CH_3_CH_2_)_3_S]AgBiI_5_ were grown following a previously reported procedure.^[^ ^29^ ^]^ (CH_3_CH_2_)_3_SI (1 Eq.) and I_2_ (2 Eq.) powders were scaled into a 4.5 ml vial and left for 1 min to form a melt. AgI (0.2 Eq.) and BiI_3_ (0.2 Eq.) powders were subsequently added to the mixture and placed on a shaker at 80 °C for 30 min, after which it was placed at an undisturbed spot. After ≈5 months, red rhombohedral crystals with dimensions of ≈ 5 × 4 × 1 mm^3^ were hand‐picked for subsequent X‐ray detector characterization.

### X‐ray Powder Diffraction

Powder X‐ray diffraction (PXRD) data of the powders of [(CH_3_CH_2_)_3_S]_6_Bi_8_I_30_ and [(CH_3_CH_2_)_3_S]AgBiI_5_ was collected in a Bragg‐Brentano‐Geometry with a D8 ADVANCE Diffractometer (Bruker AXS, Germany) using Cu−K_α_ radiation (1.54178 Å) with a LYNXEYE XE‐T detector. Diffraction data was recorded at 40 kV and 40 mA with a step size of 0.02° and measurement time of 0.5 s per step. The PXRD measurements were conducted on PMMA and PCV sample holders.

### SEM Characterization

Scanning Electron Microscopy (SEM) analysis was conducted using a Zeiss Ultra Plus SEM equipped with a Gemini column. The following steps outline the specific conditions and settings employed during the analysis. Samples were mounted on aluminum stubs using conductive carbon tape to ensure proper conductivity and adhesion. Imaging was performed at an acceleration voltage of 3 kV. Secondary Electron (SE) detection mode was employed to obtain high‐resolution images, providing detailed surface morphology insights (Figure S9 and S10, Supporting Information). A bias of 300 V was applied during the imaging process to enhance image quality. The working distance was varied based on imaging requirements, with a standard setting of 8 mm for surface imaging to optimize resolution and focus.

### X‐ray Fluorescence Analysis

X‐ray fluorescence (XRF) measurements were performed on compressed pellet samples of ≈1.2 mm thickness of the target compounds using a PANalytical MagiX PRO XRF spectrometer (Malvern Panalytical Ltd., Malvern, UK) equipped with a Rh anode X‐ray tube. Standardless elemental analysis was carried out using the Omnian 10 mm program to estimate the elemental weight percentages.

### Optoelectronic Characterization

Diffuse reflectance spectroscopy was applied to the powders of [(CH_3_CH_2_)_3_S]_6_Bi_8_I_30_ and [(CH_3_CH_2_)_3_S]AgBiI_5_ to determine the bandgap of the materials. The optical properties were recorded over a spectral range from 300–1100 nm employing an Avantes AvaSpec‐2048 dual UV–vis spectrophotometer, which includes an integrating sphere with an integrated light source. The recorded reflectance data was subsequently converted to absorption equivalents by applying the Kubelka–Munk function, *k*/*s* = (1 – *R*)^2^/2*R* (*k* = 4π*κ*/*λ* is the absorption coefficient of the sample, *s* is the scattering coefficient, *R* is the diffuse reflectance, and kappa the extinction coefficient).^[^ ^45^ ^]^ To estimate the optical bandgap, *E*_g_, of the materials, the Kubelka‐Munk function was extrapolated to *k* = 0 to yield an approximate value of it (Figure S1, Supporting Information).

### Device Fabrication and Characterization

The 1‐2‐mm‐thick [(CH_3_CH_2_)_3_S]_6_Bi_8_I_30_ and [(CH_3_CH_2_)_3_S]AgBiI_5_ pellets were employed for detector assembly. The Au electrodes (area of 0.16 or 0.206 cm^2^ and thickness of ≈20 nm or ≈70 nm) were thermally evaporated through a shadow mask on the opposite faces of the compressed pellets under a vacuum of ≈10⁻⁶ bar. It is important to note that the attenuation depth for 8 keV photons in gold is approximately 2.5 µm, which means the percentage of intensity loss of 8 keV X‐rays in the ≈20 and 70 nm gold contact layers is ≈0.8% and 2.8%, respectively. Therefore, the attenuation in the gold contacts can be disregarded. A Keithley 2400 Source Meter was used to apply the bias voltage and record the response current. All Characterization was conducted at room temperature in air with optical and electrical shielding to eliminate the influence of electromagnetic and ambient light.

### X‐ray Detector Characterization

X‐ray response curves were obtained using a Cu anode X‐ray source with a primary Kα emission line at 8.05 keV, integrated into an Empyrean Series 3 X‐ray diffractometer from Malvern Panalytical. The X‐ray tube was operated at 40 kV and 40 mA, positioned perpendicular to the compressed pellet. Dose rate calibration was performed with a dosimeter (STEP OD‐01, Pockau, Germany) to ensure accurate measurements. To modulate the X‐ray dose rates, a series of attenuation filters with varying thicknesses (0.1 mm Cu, 0.2 mm Cu, 0.3 mm Cu, and 0.4 mm Cu+0.02 mm Ni) were placed between the X‐ray source and the sample (Figure S4; Table S2, Supporting Information), the distance between the source and detector is fixed. The dose rate, *D*, has been evaluated with a dosimeter (STEP OD‐01, Pockau, Germany). The ionizing radiation dose in air, *D_air_*, was then calculated as

(1) $$D_{air}=\frac{D\cdot A}{k\cdot T_{07}}\cdot \frac{{\left(\mu /\rho \right)}_{air}}{{\left(\mu /\rho \right)}_{tissue}}\approx D\cdot A\cdot 1.286\left(\frac{Gy}{s}\right)$$

where *A* is the contact area, (*µ*/*ρ*)*_air_ *and (*µ*/*ρ*)*_tissue_ *are the mass attenuation coefficients of 8 keV photons in air and soft tissue, respectively, *k *is relative energy‐dependent response of OD‐01 detector to 8 keV, *T_07_ *is the transmittance of photons with energy 8 keV in 0.07 mm soft tissue.^[^ ^59^ ^]^

Additional X‐ray measurements were carried out at the KMC‐3 XPP beamline of the BESSY II synchrotron (Helmholtz‐Zentrum Berlin) using photon energies of 8 and 12 keV. The photon flux was measured with a Hybrid Photon Counting detector (DECTRIS PILATUS 100K) and adjusted by combining aluminum absorbers of thicknesses ranging from 50 to 900 µm (Figure S5; Table S3, Supporting Information). Despite operating in the standard mode (known as multibunch hybrid mode operation), where X‐ray timing is determined by the charge distribution of short electron bunches circulating in the storage ring at 2 ns (60 cm) intervals and bunch lengths ranging from 45 to 80 ps (rms),^[^ ^60^ ^]^ the detectors under study demonstrated a reliable response.

For both measurement setups, a Keithley 2400 Source Meter was employed to apply the bias voltage and record the photocurrent. A Labview software script that we developed allowed the built‐in voltage‐source of the electrometer to be varied from +200 V to −200 V in steps. The collected voltage‐current characteristics, the geometric dimensions of the contacts and the geometric dimensions of the samples were used to determine the resistivity. All characterizations were conducted at room temperature and in ambient air, with optical and electrical shielding to minimize the impact of electromagnetic interference and ambient light.

The ability of carrier extraction was determined by the mobility‐lifetime (µ*τ*) product, which can be derived by fitting the signal current, derived by subtracting the average dark current from the corresponding average photocurrent, using a modified Hecht Equation (2):^[^ ^9^, ^40^, ^49^ ^]^

(2) $$I_{signal}=I_{0}\frac{\mu \tau V}{d^{2}}\cdot \left(1-exp\left(-\frac{d^{2}}{\mu \tau V}\right)\right)$$

where *I_0_* is the saturated current, *V* is the applied voltage, *d* is the detector thickness, *µ* and *τ* are the charge carrier mobility and lifetime, respectively.

The X‐ray sensitivity, *S*, is defined as the signal current density per unit radiation exposure, and is given by:^[^ ^30^, ^49^, ^61^, ^62^ ^]^

(3) $$S=\frac{{\bar{I}}_{photo}-{\bar{I}}_{dark}}{A\cdot D_{air}}=\frac{I_{signal}}{A\cdot D_{air}}$$

where *A* is the electrode area, and *D_air_* is the X‐ray dose rate. The maximum theoretical sensitivity (*S_0_* in µC Gy_air_^−1^ cm^−2^), assuming no photoconductive gain, is described by the following Equation (4):^[^ ^9^, ^53^ ^]^

(4) $$S_{0}=\left(\frac{q\cdot 6.21\cdot 10^{21}}{\left(\alpha_{air}/\rho_{air}\right)\cdot W}\right)\;\cdot \left(\frac{\alpha_{en}}{\alpha }\right)$$

where *q* is the electron charge, *α_air_* and *ρ_air_* are the energy absorption coefficient and density of air, *α_en_* and *α* are the energy absorption and linear attenuation coefficients of the detector material, and *W* is the electron‐hole pair creation energy. The value of *W* is calculated as *W*(eV) = 2.8*E_g_* + 0.5,^[^ ^63^ ^]^ resulting in *W* = 6.1 eV for [(CH_3_CH_2_)_3_S]_6_Bi_8_I_30_ and *W* = 5.54 eV for [(CH_3_CH_2_)_3_S]AgBiI_5_.

The **limit of detection (LoD)** is defined as the dose rate at which a **signal‐to‐noise ratio (SNR) of 3** is achieved at a given voltage. The SNR is calculated as follows:^[^ ^8^, ^61^ ^]^

(5) $$\mathrm{SNR}\;=\frac{I_{signal}}{I_{noise}}=\frac{{\bar{I}}_{photo}-{\bar{I}}_{dark}}{\sqrt{\frac{1}{N}\sum_{i}^{N}{\left(I_{i}-{\bar{I}}_{photo}\right)}^{2}}}$$

The dark current drift is calculated as:^[^ ^7^, ^21^ ^]^

(6) $$\Delta I\;=\frac{I_{finish\;}-\;I_{start}}{tAV/d}$$

where *I_finish_* and *I_start_* are the final and initial current, *t* is the measurement duration, *A* is the electrode area, *d* is the detector thickness, and *V* is the applied bias voltage.

### Statistical Analysis

All data processing, visualization, and fitting were performed using OriginPro 2021b (OriginLab Corporation, Northampton, MA, USA). No additional statistical tests were applied, as the analysis primarily involved averaging multiple measurements and extracting parameters from curve linear and exponential fitting routines provided in OriginPro. Sample‐to‐sample variation in resistivity, sensitivity, and limit of detection is presented both as a range and as mean values with statistical error, with the number of samples specified in the text.

## Conflict of Interest

The authors declare no conflict of interest.

## Supporting information



Supporting Information

## Data Availability

The data that support the findings of this study are available from the corresponding author upon reasonable request.
