# Supplementary material for: Green Fabrication of Sulfonium‐Containing Bismuth Materials for High‐Sensitivity X‐Ray Detection
Source: Adv Mater. 2025 Apr 10;37(24):2418626. doi: 10.1002/adma.202418626 (PMC12177852; doi:10.1002/adma.202418626)
Supplement: Supplementary file 1 — Supporting Information [file ADMA-37-2418626-s001.pdf]

ADVANCED MATERIALS

Supporting Information

for *Adv. Mater.*, DOI 10.1002/adma.202418626

Green Fabrication of Sulfonium-Containing Bismuth Materials for High-Sensitivity X-Ray Detection

*Allan Starkholm, Dominik Al-Sabbagh, Sema Sarisozen, Alexander von Reppert, Matthias Rössle, Markus Ostermann, Eva Unger, Franziska Emmerling, Lars Kloo, Per H. Svensson, Felix Lang and Olena Maslyanchuk**

Supporting Information
for

Green Fabrication of Sulfonium-Containing Bismuth Materials for High-Sensitivity X-ray Detection

Allan Starkholm[1], Dominik Al-Sabbagh[2], Sema Sarisozen[3], Alexander von Reppert[4], Matthias Rössle[5], Markus Ostermann[7], Eva Unger[1], Franziska Emmerling[2], Lars Kloo[6], Per H. Svensson[6], Felix Lang[3], Olena Maslyanchuk[1†]

[1]*Department Solution-Processing of Hybrid Materials and Devices, Helmholtz-Zentrum Berlin, 14109 Germany*

[2]*Department of Materials Chemistry, Federal Institute for Materials Research and Testing, Berlin, 12205 Germany*

[3]*Freigeist Juniorgroup, Radiation Tolerant Electronics with Soft Semiconductors (ROSI), Universität Potsdam, Institut für Physik und Astronomie, Physik weicher Materie, Potsdam-Golm, 14476 Germany*

[4]*Soft Matter Physics and Optoelectronics Group, Universität Potsdam, Institut für Physik und Astronomie, Physik und Optoelektronik weicher Materie, Potsdam-Golm, 14476 Germany*

[5]*Research Group Ultrafast Dynamics, Helmholtz-Zentrum Berlin, 14109 Germany*

[6]*Applied Physical Chemistry, Department of Chemistry, KTH Royal Institute of Technology, Stockholm SE-114 28, Sweden*

[7]*Department of Process Analytical Technology, Federal Institute for Materials Research and Testing, Berlin, 12205 Germany*

[†] olena.maslyanchuk@helmholtz-berlin.de

Contents

Table S1. Performance of X-ray detectors based on materials investigated as compressed pellets............3
Fig. S1. The Kubelka-Munk absorption curves for the powders 4
Supplementary Note 1. Electrical and photoelectric properties of the X-ray detectors 4
Fig. S2. Electrical and photoelectric properties of the X-ray detectors 5
Supplementary Note 2. X-ray absorption...6
Fig. S3. X-ray absorption..7
Supplementary Note 3. X-ray response experiments 7
Fig. S4. Image and schematic of the X-ray response measurement setup housed within the Empyrean Series 3 X-ray diffractometer (University of Potsdam) 7
Table S2. Copper absorber thickness and corresponding dose rates in the chamber of XRD setup. 7
Fig. S5. Image of the X-ray response measurement setup housed at the KMC-3 XPP beamline at the synchrotron source BESSY II (Helmholtz-Zentrum Berlin)...8
Table S3. Aluminium absorber thickness and corresponding attenuation factor of 8 keV and 12 keV X-ray photon energy at the KMC-3 XPP beamline at the synchrotron source BESSY II (HZB)................................8
Fig. S6. Performance of the compressed pellet X-ray detectors..9
Supplementary Note 4. Sensitivity and photoconductive gain in the X-ray detector devices....................10
Fig. S7. Calculated maximum theoretical sensitivity..10
Supplementary Note 5. SCLC measurements. 11
Fig. S8. SCLC curves 11
Supplementary Note 6. Temperature-dependent conductivity 12
Fig. S9. Temperature-dependent conductivity...12
Fig. S10. X-ray dose rate dependent signal-to-noise ratio 13
Fig. S11. Bias dependent photocurrent..14
Fig. S12. Aging bar charts of the sensitivity (a) and limit of detection..14
Fig. S13. Irradiation- and bias stability after six more month of storage...15
Supplementary Note 7. Scanning electron microscopy images...16
Supplementary Note 8. X-ray fluorescence data 18
References...19

Table S1. Performance of X-ray detectors based on materials investigated as compressed pellets.

No	Detector structure	Methods of preparation	Resistivity (GΩ cm)	$\mu\tau$ product, $\times10^{-3}$ ($cm^2 V^{-1}$)	Electric field ($V mm^{-1}$)	Sensitivity ($\mu C Gy_{air}^{-1} cm^{-2}$)	Anode acceleration voltage (X-ray energy)	Detection limit ($nGy_{air} s^{-1}$)	Year	Ref.
1	Ag/ZnO/PCBM/$MAPbI_3$/<br>PEDOT:PSS/ITO/Glass	Precipitation reation+pressing	NA	0.2	200	2 527	W 70 kV<br>(38 keV)	48 000	2017	[1]
2	Au/$Cs_2AgBiBr_6$/Au	Solution grown+ball-milling+pressing+annealing	14	5.51	500	250	W 50 kV<br>(30 keV)	95.3	2019	[2]
3	Au/$CsPbBr_3$/FTO	Four-step hot pressing	0.005	5.12-13.2	5	55 684	W 50 kV<br>(30 keV)	215	2019	[3]
4	Au/$MAPbI_3$/PCBM/Au	Grinding+hot pressing	NA	0.384	12.5	122 000	Ag 40 kV	NA	2020	[4]
5	Au/$MA_3Bi_2I_9$/Au	Solvent evaporation+grinding+pressing	228	0.046	70	563	W 45 kV<br>(35.5 keV)	9.3	2020	[5]
6	Cr/$Cs_3Bi_2Br_3I_6$/Pt<br>Cr/$Cs_3Bi_2I_9$/Pt<br>Cr/$Cs_2AgBiBr_6$/Pt<br>Cr/BiI_3/Pt	Solvent evaporation+ball milling+pressing+ annealing	71	0.0364<br>0.0416<br>0.0117<br>0.046	714.3<br>714.3<br>714.3<br>285	2.5<br>1.5<br>7<br>50	W 70 kV	10 700<br>29 700<br>20 300<br>5 500	2021	[6]
7	Cr/$MAPbI_3$/Au	Pressing+hot pressing	2.4	0.4	30	9 300	W 70 kV<br>(52 keV)	6.3	2021	[7]
8	Au/Ba_2AgIO_6/Au	Solution grown+pressing			5	18.9	Au 50 kV<br>(50 keV)	N/A	2021	[8]
9	Au/$MAPbI_3$/Au	Solvent evaporation +hot pressing+annealing		5.46	6.58	420 000	Ag 40 kV<br>(40 keV)	350	2021	[9]
10	Au/$(MA_{0.7}Cs_{0.3})Bi_2I_9$/Au	Anti-solv.-ass. synthesis+ pressing+annealing	190	10^{-3}-10^{-4}	128.6	130	W 50 kV<br>(40 keV)	12.8	2022	[10]
11	Au/$MAPbI_3$/Au	Solution grown+ pressing+annealing			5	169	Au 40 kV<br>(40 keV)	N/A	2022	[11]
12	Au/$Cs_3Bi_2I_9$/Au	Ball-milling+hot pressing	13.3	0.167	40	230.46	Ag 40 kV	61.25	2022	[12]
13	Au/$Cs_3Bi_2I_9$/Au	Anti-solvent-assist.+pressing+Ball-milling+pressing	0.513<br>2.21		28.8<br>28.8	588<br>543	W	76<br>155	2022	[13]
14	Au/(F-PEA)$_3$$BiI_6$/$C_{60}$/BCP/Cr	Solution grown +pressing	50-200	0.083-0.4	100	52.6-118.6	120 keV	30-1800	2022	[14]
15	Au/<br>BA_2PbI_4/$MAPbI_3$/Au	Solvent evaporation +hot pressing	8.5	1.18	12.5	2 000	Ag<br>40 kV	111.76	2022	[15]
16	Au/PCBM/$MAPbI_3$/Au	Anti-solvent-assist.+hot pressing+annealing	1.43	0.87	50	15 800	50 kV	410	2023	[16]
17	Au/PCBM/$CsPbBr_3$/Au	Ball-milling+pressing+annealing	0.316	0.516	100	14 430		564	2023	[17]
18	Au/$BiVO_4$/Au	Precipitation+pressing+annealing	1300	0.175	40	241.3	Ag 40 kV	62	2023	[18]
19	Au/$MAPbI_3$/Au	Solution grown+grining+hot pressing		0.344		8291.22	Ag<br>50 keV	340	2023	[19]
20	Ag/$CsPbBr_3$-$CsPb_2Br_5$-<br>$CsPbI_xBr_{3-x}$/Ag	Coprecipitation+pressing +spray coating+annealing	1.3	1.01	18	20 555.1	Ag 45 kV	127.7	2024	[20]
21	Au/$[(CH_3CH_2)_3S]_6Bi_8I_{30}$/<br>Au<br>Au/$[(CH_3CH_2)_3S]AgBiI_5$/<br>Au	Ball-milling+pressing	11.8<br>31.0	0.152<br>0.156	120<br>120	15 190<br>14 100	Cu 40 kV	90<br>78		This work

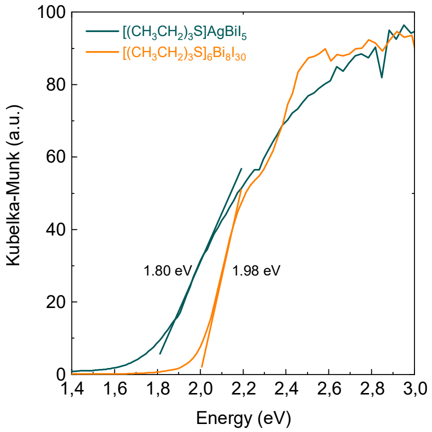

Fig. S1. The Kubelka-Munk absorption curves for the powders of $[(CH_3CH_2)_3S]AgBiI_5$ and $[(CH_3CH_2)_3S]_6Bi_8I_{30}$.

Supplementary Note 1. Electrical and photoelectric properties of the X-ray detectors of $[(CH_3CH_2)_3S]AgBiI_5$ and $[(CH_3CH_2)_3S]_6Bi_8I_{30}$.

The room temperature current-voltage (*I*-*V*) characteristics of the Au/$[(CH_3CH_2)_3S]_6Bi_8I_{30}$/Au and Au/$[(CH_3CH_2)_3S]AgBiI_5$/Au X-ray detectors were determined in the dark and under X-ray in a wide voltage range (0±200 V) by forward (FW) and backwards (BW) scans (Fig. S2a). It should be noted that the hysteresis observed at high scan speeds is weaker at low scan speeds. It is explained by the presence of mobile ionic species that may originate from vacancies or interstitials in the perovskite pellets.[21] The applied bias during the forward voltage scan results in the drift of charged mobile ions to the corresponding electrodes, which leads to the emergence of an internal electric field into the pellet. When the scanning direction is changed, the system is still under “pre-bias effect” conditions, and consequently, mobile ions are still displaced toward the electrode interfaces.[22] As the mobile ions require a certain diffusion time to migrate back to their initial position, the hysteresis is sufficiently weak at low scan speeds. Additionally, the hysteresis observed in the dark becomes even weaker under X-ray irradiation due to the increased concentration of charge carriers. The FW/BW current ratio for high scan speed is greater than 1±10% (Fig. S2b, shaded area) in the whole range of bias voltage in dark and at $V < 125$ V under X-ray irradiation (Fig. S2b, dotted lines). In contrast, for low scan speed, it achieves 1±10% at $V \approx 125$ V in dark and under a dose rate of 15.42 $\mu Gy_{air}\ s^{1}$ and 0.321 $\mu Gy_{air}\ s^{1}$ at $V \approx 25$ V. The higher the dose rate (and the concentration of photoinduced charge carriers), the lower the contribution of the ionic component in the mixed electronic-ionic conductivity.

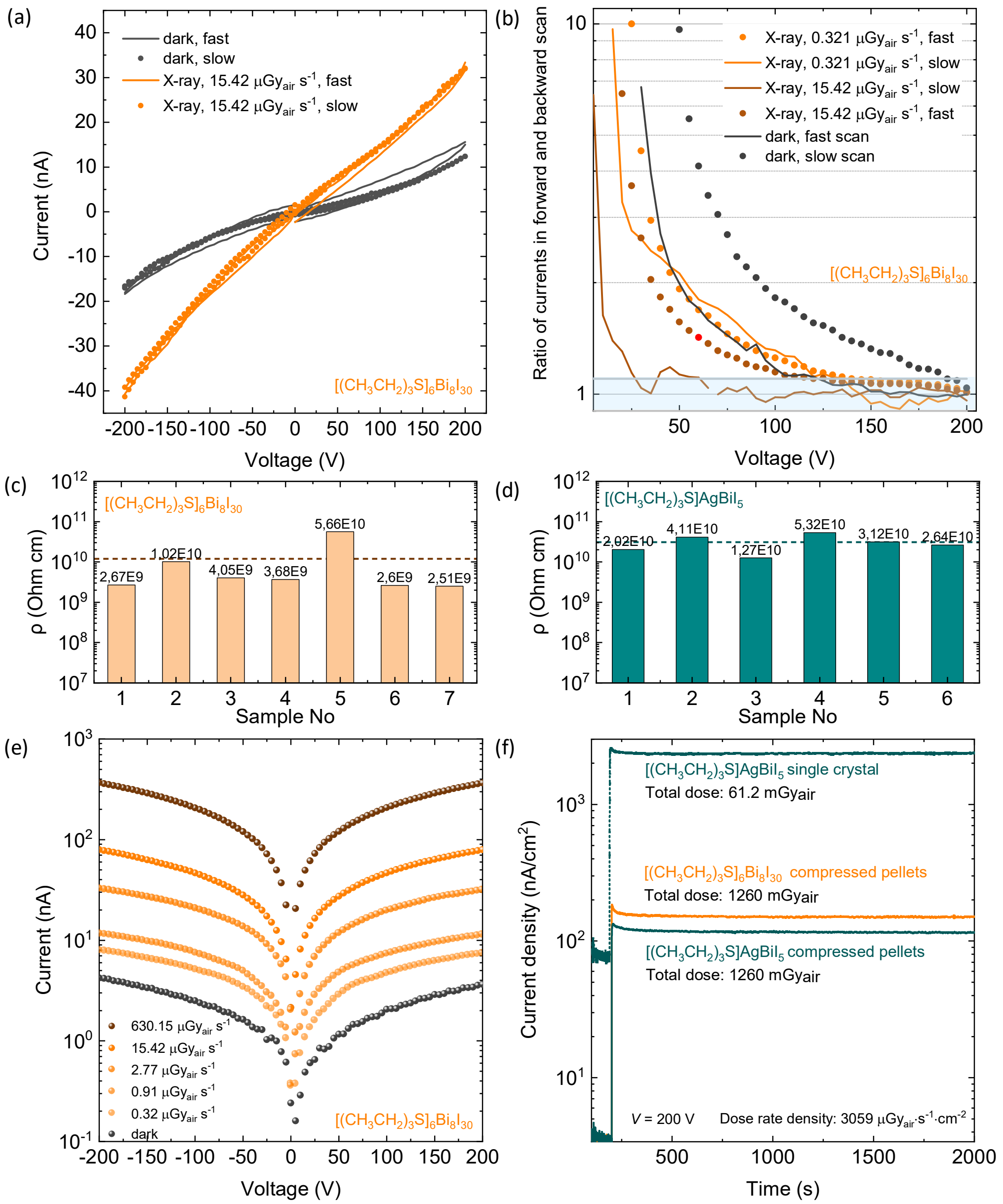

Fig. S2. Electrical and photoelectric properties of the X-ray detectors of $[(CH_3CH_2)_3S]AgBiI_5$ and $[(CH_3CH_2)_3S]_6Bi_8I_{30}$. a) Time-dependent hysteresis between forward and backward scans of the room temperature current-voltage characteristics of an Au/$[(CH_3CH_2)_3S]_6Bi_8I_{30}$/Au detector measured in the dark and under an X-ray dose rate of 203.31 μGy_{air} s^{-1} at high scan speeds (settling time 60 ms, ~35.46 V s^{-1}) (blue lines) and at low scan speeds (settling time 1 s, ~4.625 V s^{-1}) (orange lines); b) Voltage dependence of the ratio of currents in forward and backward scan under the X-ray dose rates of 15.42 μGy_{air} s^{-1} and 0.321 μGy_{air} s^{-1} and in dark with 60 ms settling time (dashed lines) and 1000 ms settling time (solid lines); c) and d) Bar charts of the resistivity of two sets of $[(CH_3CH_2)_3S]AgBiI_5$ and $[(CH_3CH_2)_3S]_6Bi_8I_{30}$ compressed pellet detectors. The mean values of resistivity are shown by dotted lines; e) *I–V* curves of an Au/$[(CH_3CH_2)_3S]_6Bi_8I_{30}$/Au detector recorded in the dark and under X-ray irradiation at the different dose rates. It shows a distinct increase in photocurrent as the dose rate increases; f) The long-term current response of the X-ray detector devices under a 200 V bias voltage and 2000 s X-ray illumination, measured for $[(CH_3CH_2)_3S]_6Bi_8I_{30}$ and $[(CH_3CH_2)_3S]AgBiI_5$ detectors based on compressed pellets (D_{air} = 630.15 μGy_{air} s^{-1}), as well as for a SC detector of $[(CH_3CH_2)_3S]AgBiI_5$ (D_{air} = 30.59 μGy_{air} s^{-1}).

Supplementary Note 2. X-ray absorption of $[(CH_3CH_2)_3S]AgBiI_5$ and $[(CH_3CH_2)_3S]_6Bi_8I_{30}$.

The X-ray absorption and charge collection efficiency of $[(CH_3CH_2)_3S]AgBiI_5$ and $[(CH_3CH_2)_3S]_6Bi_8I_{30}$ are critical to understanding the performance of these detectors in X-ray detection applications. Both materials exhibit high absorption efficiency due to the presence of heavy elements such as bismuth (Bi) and iodine (I), which have high atomic numbers that facilitate effective attenuation of X-rays, especially in the energy range relevant to medical imaging and industrial inspection. In order to determine the X-ray absorption, the attenuation coefficients of $[(CH_3CH_2)_3S]_6Bi_8I_{30}$ and $[(CH_3CH_2)_3S]AgBiI_5$ were estimated using the photon cross-section database[23] and the densities of the materials.

The density of the $[(CH_3CH_2)_3S]_6Bi_8I_{30}$ and $[(CH_3CH_2)_3S]AgBiI_5$ compressed pellets is determined by their weight and geometrical dimensions, the latter of which was determined with a calliper. It is worth noting that the determined densities of the compressed pellets of $[(CH_3CH_2)_3S]AgBiI_5$ are very close to the theoretical value of 3.58 g·cm^3 estimated from its lattice parameters.[24]

The attenuation coefficients are presented in Figure S3a. For comparison, curves for stabilised a-Se, Si, and CdTe were also evaluated. It is worth noting that the absorption coefficients of $[(CH_3CH_2)_3S]_6Bi_8I_{30}$ and $[(CH_3CH_2)_3S]AgBiI_5$ differ only to a minor extent, with the curves in Fig. S3a being nearly identical across the entire energy range and in Fig. S3b aligning closely at 10 keV. It is evident that the stopping power of $[(CH_3CH_2)_3S]_6Bi_8I_{30}$ and $[(CH_3CH_2)_3S]AgBiI_5$ is significantly higher than that of Si and is comparable to a-Se and CdTe in the photon energy range of 1-100 keV commonly used for X-ray medical imaging (Fig. S3a). The fraction of incident photons of the beam that is attenuated by the photoconductor, called the attenuation efficiency, depends on the linear attenuation coefficient α of the photoconductor material and its thickness d. This can be calculated using the equation $A_Q = 1 - \exp^{(-\alpha d)}$. The low energy photons (10 keV) are completely absorbed (A_Q = 99.9%) in 0.08 mm, 0.15 mm, 0.35 mm and 0.88 mm thick layers of CdTe, $[(CH_3CH_2)_3S]AgBiI_5$, a-Se, and Si, respectively, as shown in Fig. S3b. There is a noticeable difference in attenuation capacity in the high-energy range. For example, a 1-mm-thick $[(CH_3CH_2)_3S]AgBiI_5$ compressed pellet attenuates 57% of the incident 100 keV X-ray photons, as compared to 64%, 25% and 4% for CdTe, a-Se and Si, respectively (Fig. S3b).

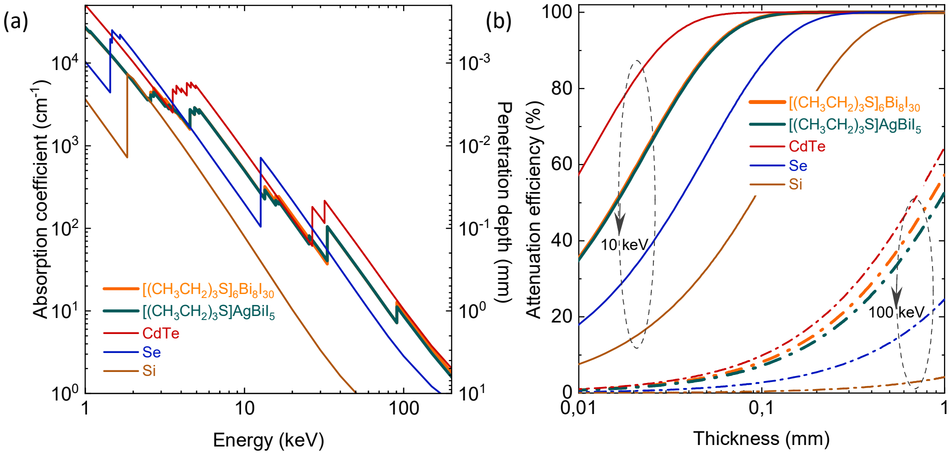

Fig. S3. X-ray absorption $[(CH_3CH_2)_3S]AgBiI_5$ and $[(CH_3CH_2)_3S]_6Bi_8I_{30}$. a) Linear attenuation coefficients and attenuation depths of a-Se, Si, CdTe, $[(CH_3CH_2)_3S]_6Bi_8I_{30}$ and $[(CH_3CH_2)_3S]AgBiI_5$ as a function of photon energy. b) The attenuation efficiency versus thickness of a few representative semiconductor materials exposed to 10 and 100 keV X-ray photons.

Supplementary Note 3. X-ray response experiments

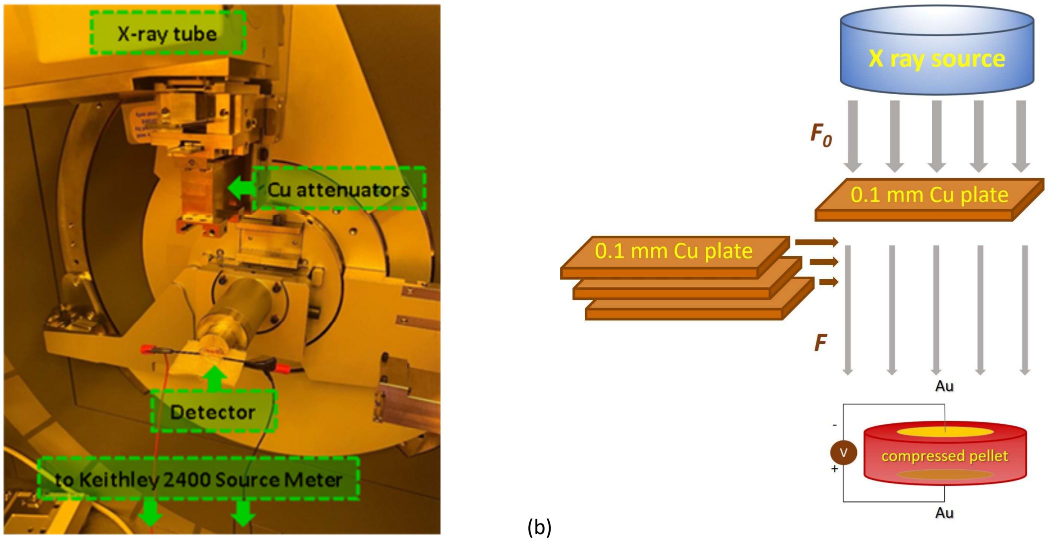

Fig. S4. Image and schematic of the X-ray response measurement setup housed within the Empyrean Series 3 X-ray diffractometer (University of Potsdam).

Table S2. Copper absorber thickness and corresponding dose rates in the chamber of XRD setup.

No	Copper absorber thickness, mm	Measured dose rate D, µGy s^{-1} cm^{-2}	Calculated by dose rate D_{air}/A, µGy s^{-1} cm^{-2}
1	0	2379.376	3059.418
2	0.1	58.119	74.730
3	0.2	10.47	13.462
4	0.3	3.418	4.395
5	0.4	1.21	1.556

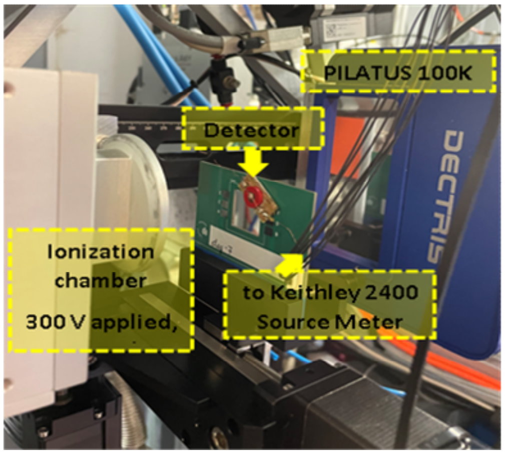

Fig. S5. Image of the X-ray response measurement setup housed at the KMC-3 XPP beamline at the synchrotron source BESSY II (Helmholtz-Zentrum Berlin). The radiation dose rate was adjusted by sequential insertion of Al plates with thicknesses from 50 to 900 µm (Table S3). A Keithley 2400 Source Meter was used to apply the bias voltage and record the response current. All characterization was conducted at room temperature in air with optical and electrical shielding to eliminate the influence of electromagnetic and ambient light.

Table S3. Aluminium absorber thickness and corresponding attenuation factor of 8 keV and 12 keV X-ray photon energy at the KMC-3 XPP beamline at the synchrotron source BESSY II (HZB).

Aluminium absorber thickness, µm	Attenuation factor @ 8 keV	Attenuation factor @ 12 keV
0	1	1
50	2.02	1.27
100	4.12	1.6
150	8.32	2.03
250	33.4	2.99
300	67.47	3.8
350	137.61	4.78
400	277.97	6.08
500	1115.56	8.83
550	2253.43	11.21
600	4596.11	14.13
650	9284.14	17.94
750	37259.7	26.4
800	75264.6	33.53
850	153509.98	42.24
900	310090.16	53.65

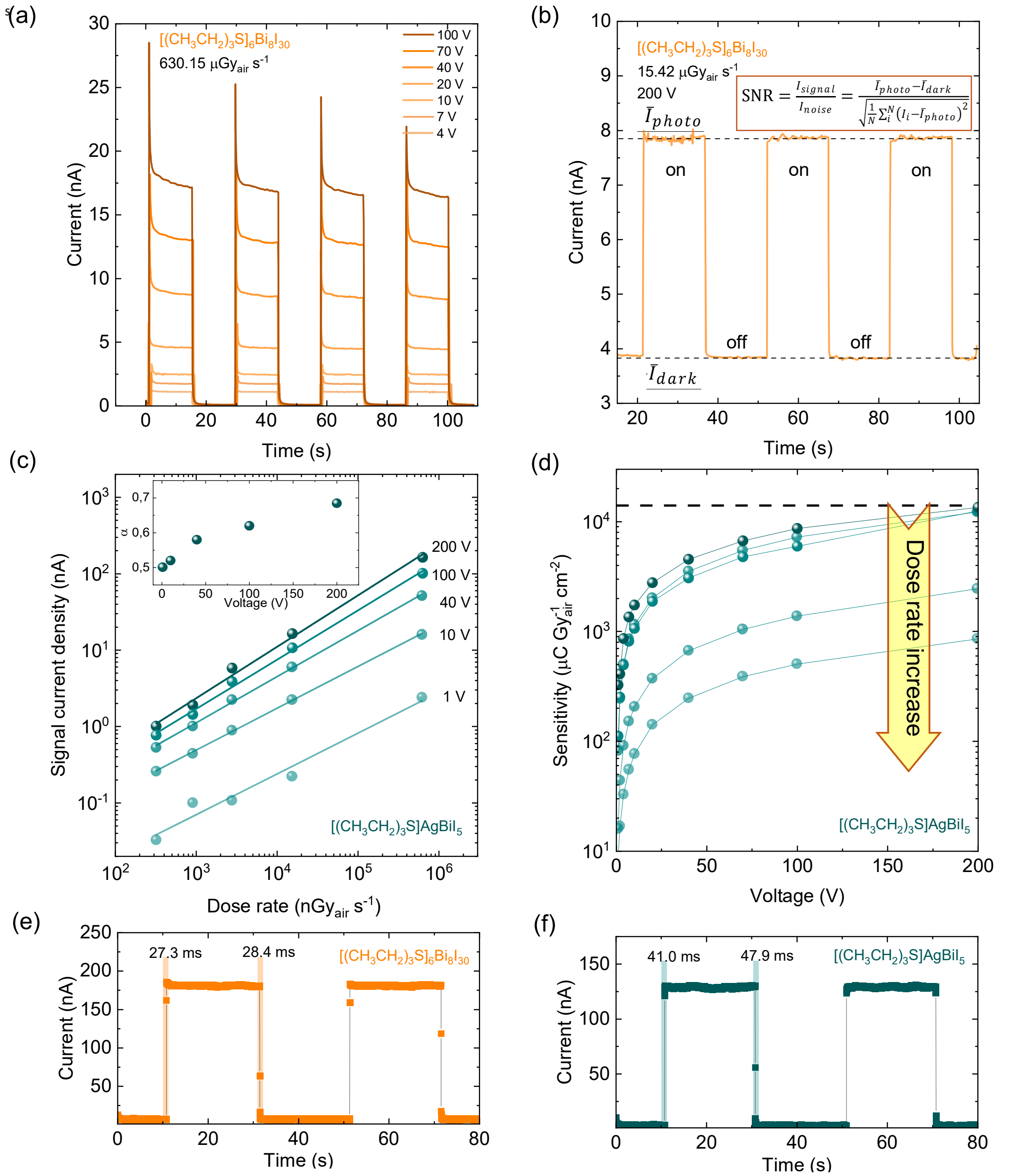

Fig. S6. Performance of the compressed pellet X-ray detectors of $[(CH_3CH_2)_3S]AgBiI_5$ and $[(CH_3CH_2)_3S]_6Bi_8I_{30}$. a) X-ray-induced current response of an Au/$[(CH_3CH_2)_3S]_6Bi_8I_{30}$/Au X-ray detector under different bias voltages at a given dose rate of 630.15 $\mu Gy_{air}\times s^{-1}$. b) X-ray photoresponse characteristics of an Au/$[(CH_3CH_2)_3S]_6Bi_8I_{30}$/Au detector with a dose rate of 489.51 $\mu Gy_{air}\times s^{-1}$ on tuning the X-ray source on and off under an electric field of 1 $V\times mm^{-1}$. The mean values of the photocurrent and the dark current are indicated by dashed lines. e) Typical dose rate dependence of signal current of an Au/$[(CH_3CH_2)_3S]AgBiI_5$/Au detector under different bias voltages. The inset shows the voltage dependence of the slope of dependences presented. Though α increases with the bias voltage, it does not reach unity due to charge carrier trapping, recombination and space-charge effects that lead to incomplete charge collection. f) Voltage dependence of the X-ray sensitivity of the detectors at different dose rates. e) and f) The X-ray photocurrent response curves at 200 V bias, under 630.15 μGy_{air} s^{-1} irradiation dose rate and an electric field of 11.1 V mm^{-1} and the derived raise time and fall time.

Supplementary Note 4. Sensitivity and photoconductive gain in the X-ray detector devices of $[(CH_3CH_2)_3S]AgBiI_5$ and $[(CH_3CH_2)_3S]_6Bi_8I_{30}$.

The high sensitivities observed in the detectors based on compressed $[(CH_3CH_2)_3S]_6Bi_8I_{30}$ and $[(CH_3CH_2)_3S]AgBiI_5$ pellets, as well as $[(CH_3CH_2)_3S]AgBiI_5$ single crystals, may partly result from photoconductive gain, similar to that reported for compressed perovskite pellets.[3,4,6,8,9,12] The maximum theoretical sensitivity (S_0 in μC Gy_{air}^{1} cm^{2}), assuming no photoconductive gain, is described by the following equation:[25–27]

$$S_0 = \left(\frac{q\cdot 6.21\cdot 10^{21}}{(\alpha_{air}/\rho_{air})\cdot W}\right)\cdot\left(\frac{\alpha_{en}}{\alpha}\right),$$

where q is the electron charge, α_{air} and ρ_{air} are the energy absorption coefficient and density of air, α_{en} and α are the energy absorption and linear attenuation coefficients of detector material, and W is the electron-hole pair creation energy. The value of W is calculated as W(eV) = $2.8E_g + 0.5$,[28] resulting in W = 6.1 eV for $[(CH_3CH_2)_3S]_6Bi_8I_{30}$ and W = 5.54 eV for $[(CH_3CH_2)_3S]AgBiI_5$. Substitution of relevant data from the photon cross-section database[23] gives at the photon energy of 8 keV (around Kα emission line of Cu anode X-ray source) the maximum theoretical sensitivity of ~17 μC Gy_{air}^{1} cm^2 for $[(CH_3CH_2)_3S]_6Bi_8I_{30}$ and 18.7 μC Gy_{air}^{1} cm^2 for $[(CH_3CH_2)_3S]AgBiI_5$, which is approximately 900 and 750 times lower than our experimental values for compressed pellets and ~40 000 times lower than that for $[(CH_3CH_2)_3S]AgBiI_5$ single crystal.

The energy range for the Cu-target X-ray tube is not strictly monochromatic. Instead, the X-ray spectrum varies with filter thickness, where the stated energy of 8.05 keV corresponds to the unfiltered Cu Kα characteristic line. As the Cu filter thickness increases, the spectrum shifts toward higher energies, effectively modifying the average photon energy of the beam.

Evaluation of the maximum theoretical sensitivity under an anode acceleration voltage of 40 kV (corresponding to the maximum possible photon energy of 40 keV) yields 2120 μC $Gyair^{-1}$ cm^{-2} for $[(CH_3CH_2)_3S]_6Bi_8I_{30}$ and 2350 μC $Gyair^{-1}$ cm^{-2} for $[(CH_3CH_2)_3S]AgBiI_5$ (Figure S7).

Thus, photoconductive gain remains significant even at high photon energies, reinforcing its role in enhancing the detector's sensitivity. Additionally, the shift in the peak energy of the attenuated X-ray beam at lower dose rates could contribute to the observed increase in sensitivity when the dose rate decreases (Figure 4b), further supporting the robustness of these materials in X-ray detection applications.

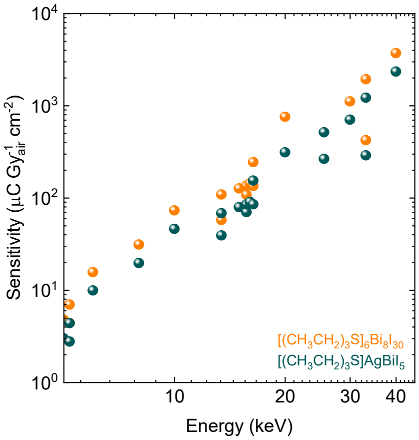

Fig. S7. Calculated maximum theoretical sensitivity of $[(CH_3CH_2)_3S]AgBiI_5$ and $[(CH_3CH_2)_3S]_6Bi_8I_{30}$ vs photon energy.

Supplementary Note 5. SCLC measurements.

To estimate the trap state density in our $[(CH_3CH_2)_3S]AgBiI_5$ and $[(CH_3CH_2)_3S]_6Bi_8I_{30}$ compressed pellets, we evaluated the trap-filled limit voltage (V_{TFL}) from dark current-voltage (*I-V*) characteristics, following the space charge limited current (SCLC) method. As depicted in Fig. S8, with increasing bias voltage, the current transitions from a linear ohmic region to the trap-filling limit (TFL) region. In the TFL region, the trap density (n_{trap}) can be calculated using the relation:

$$n_{trap} = \frac{2V_{TFL}\varepsilon\varepsilon_0}{qd^2},$$

where V_{TFL} is the trap filling limit voltage, *d* is the device thickness, *ε* is the relative dielectric constant, ε_0 is the vacuum dielectric constant, and *q* is the electron charge. Using the experimentally derived V_{TFL} values, the calculated trap density was calculated are 1.15×10^{11} cm^{-3} for the $[(CH_3CH_2)_3S]AgBiI_5$ detector and 9.82×10^{10} cm^{-3} for the $[(CH_3CH_2)_3S]_6Bi_8I_{30}$ detector. These low trap densities indicate a minimal presence of internal defects in both materials, which is promising for their performance in X-ray detection applications.

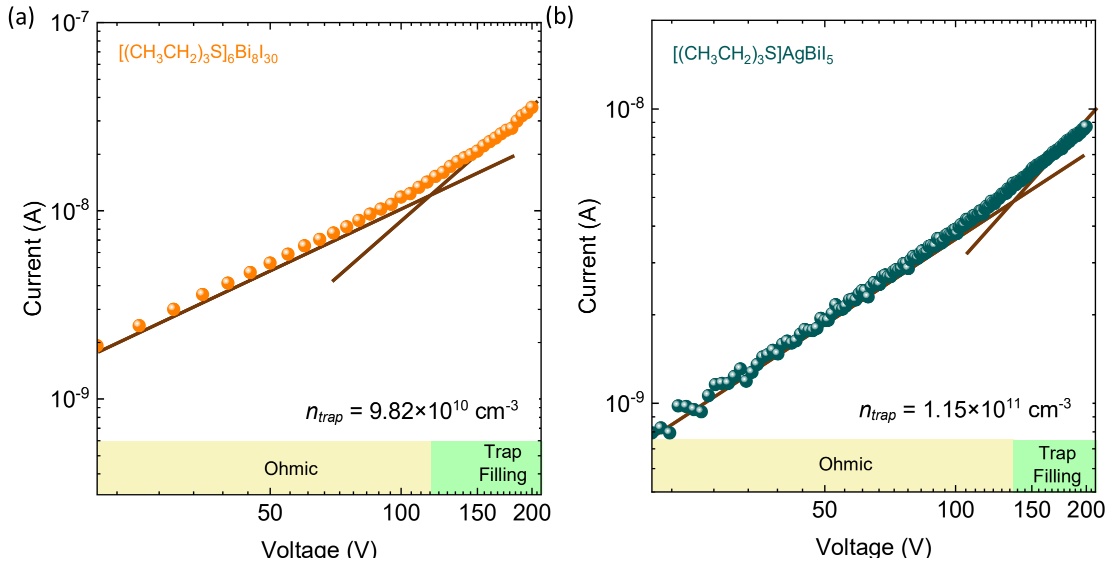

Fig. S8. SCLC curves of the $[(CH_3CH_2)_3S]AgBiI_5$ and $[(CH_3CH_2)_3S]_6Bi_8I_{30}$ detectors.

Supplementary Note 6. Temperature-dependent conductivity of the $[(CH_3CH_2)_3S]AgBiI_5$ and $[(CH_3CH_2)_3S]_6Bi_8I_{30}$ compressed detectors.

Temperature-dependent conductivity is measured to quantitatively evaluate the E_a in $[(CH_3CH_2)_3S]AgBiI_5$ and $[(CH_3CH_2)_3S]_6Bi_8I_{30}$ X-ray detectors. The conductivity in halide perovskites consists of charge and ionic conductivities. At low temperatures, ions are fixed, and the ionic conductivity is low, while when the temperature increases, the ions begin to migrate and contribute to the conductivity. The migration activation energy can be extracted from the Nernst–Einstein relation

$$\sigma(T)_0 = \left(\frac{\sigma_0}{T}\right) \exp\left(-\frac{E_a}{kT}\right),$$

where σ_0 is a constant, k is the Boltzmann constant, and T is the temperature. For $[(CH_3CH_2)_3S]AgBiI_5$ detector, the ion conductivity began to dominate above 303 K with an E_a of 0.69 eV (Fig. S9). In comparison, the conductivity of the $[(CH_3CH_2)_3S]_6Bi_8I_{30}$ device showed a transition point at 313 K with an E_a value of 0.45 eV. Both the increased transition temperature and activation energy verified the suppressed ion migration for $[(CH_3CH_2)_3S]AgBiI_5$ compressed detector; this might be ascribed to the higher grain boundary density.

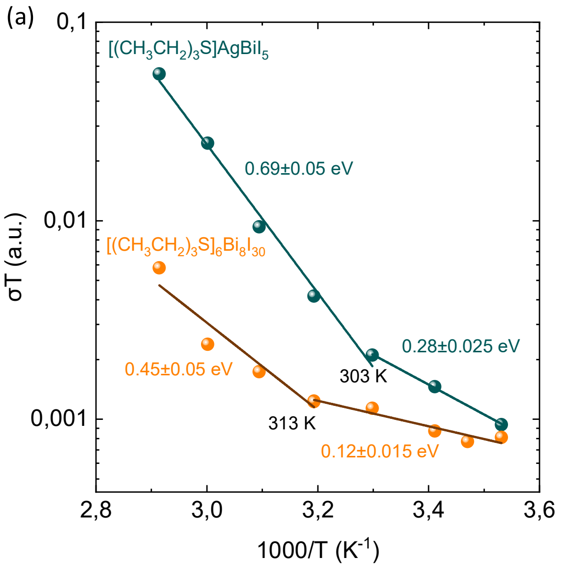

Fig. S9. Temperature-dependent conductivity of the $[(CH_3CH_2)_3S]AgBiI_5$ and $[(CH_3CH_2)_3S]_6Bi_8I_{30}$ detectors.

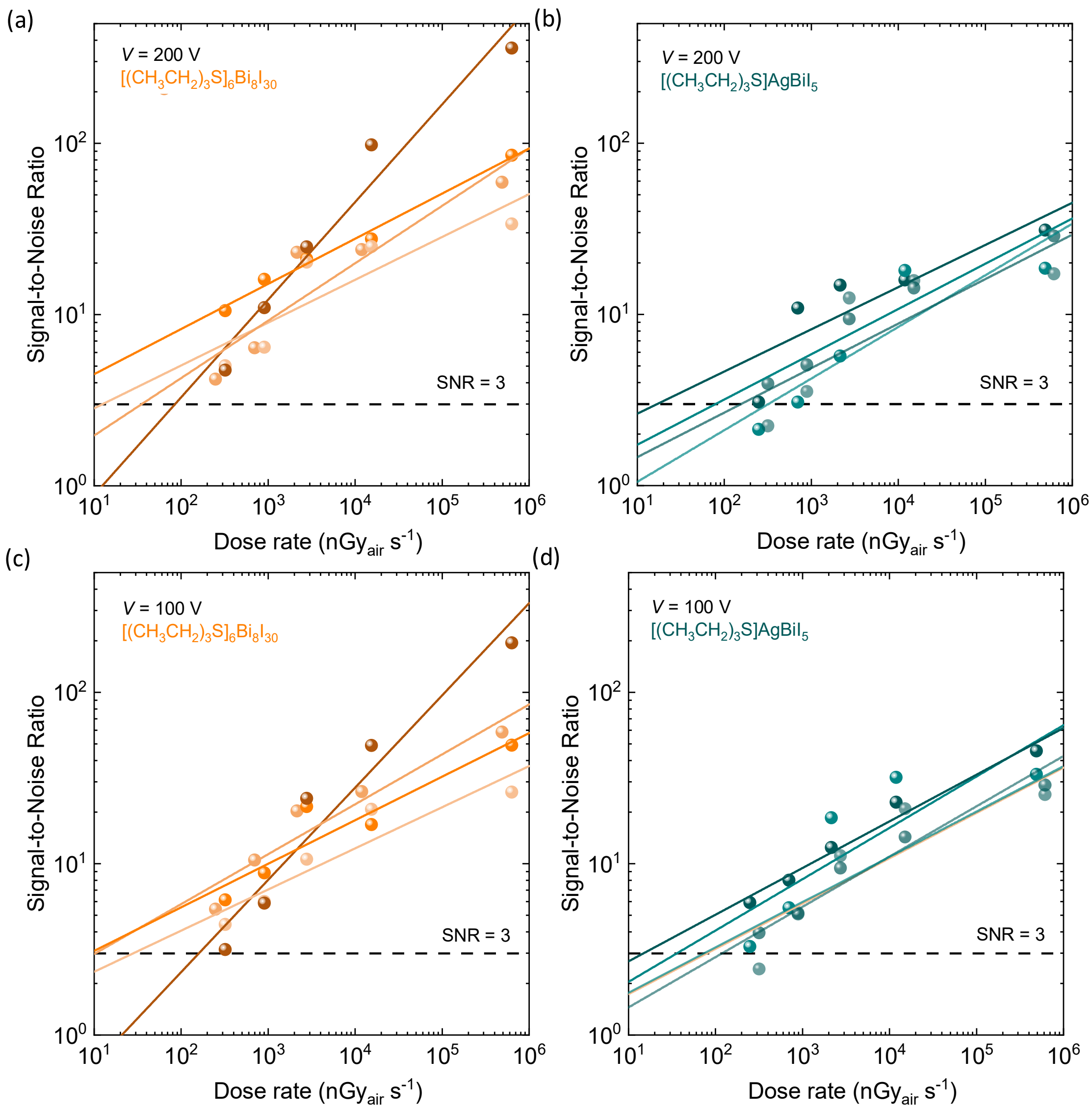

Fig. S10. X-ray dose rate dependent signal-to-noise ratio of four $[(CH_3CH_2)_3S]AgBiI_5$ (left) and $[(CH_3CH_2)_3S]_6Bi_8I_{30}$ (right) compressed pellet detectors under a bias voltage of 200 V (a,b), and 100 V (c,d). Circles show experimental results, dashed lines indicate an SNR of 3, and solid lines show the linear approximations.

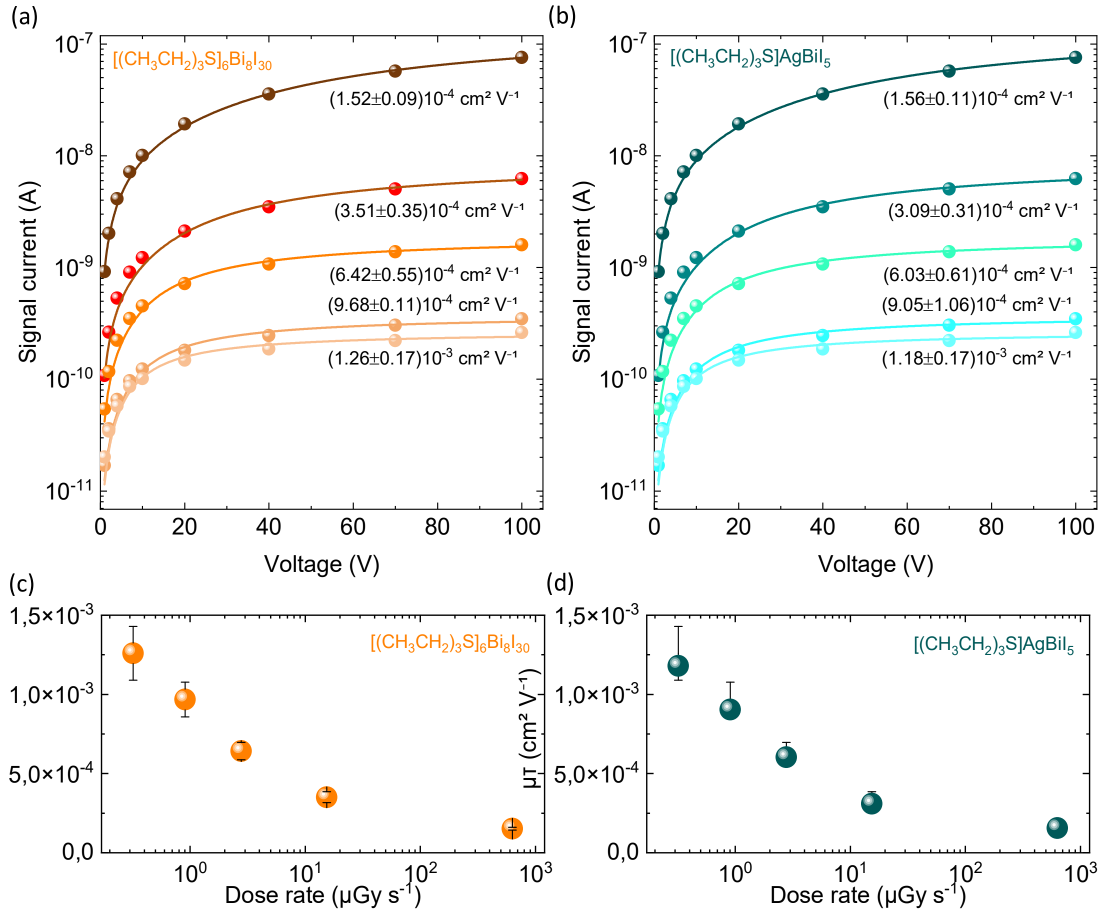

Fig. S11. Bias dependent photocurrent of the Au/[$(CH_3CH_2)_3S$]$AgBiI_5$/Au(left) and Au/[$(CH_3CH_2)_3S$]$_6Bi_8I_{30}$/Au(right) X-ray detectors under different dose rates (circles) and calculated by Hecht equation (solid lines); c, d – dose rate dependence of the derived $\mu\tau$ product.

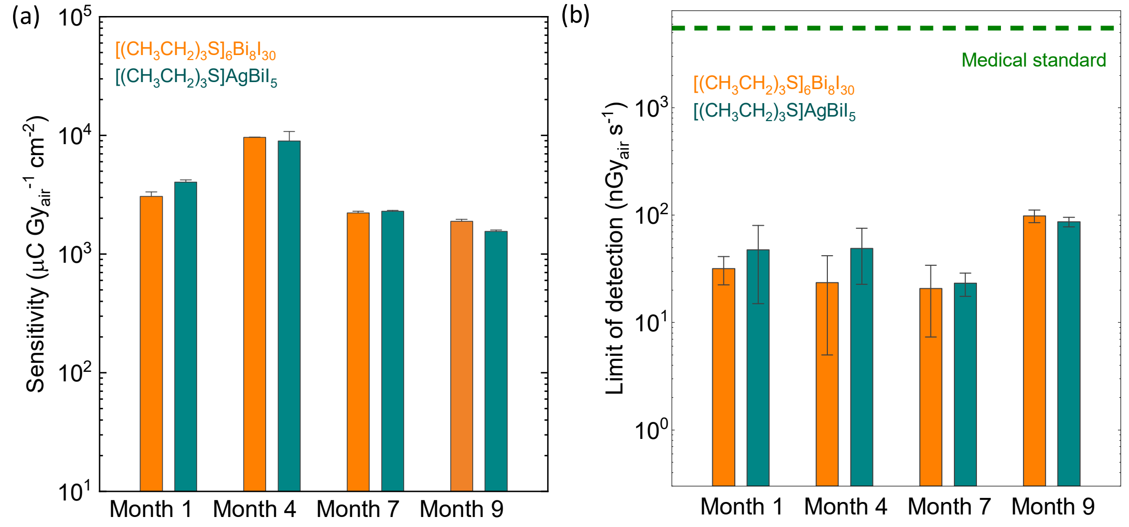

Fig. S12. Aging bar charts of the sensitivity (a) and limit of detection (b) for [$(CH_3CH_2)_3S$]$AgBiI_5$ (green) and [$(CH_3CH_2)_3S$]$_6Bi_8I_{30}$ (orange) compressed pellet detector devices under a bias voltage of 100 V. The sensitivity and LoD values are averaged over all measured devices, and the error bars represent the statistical errors. All the devices were kept in the air and under dark storage conditions.

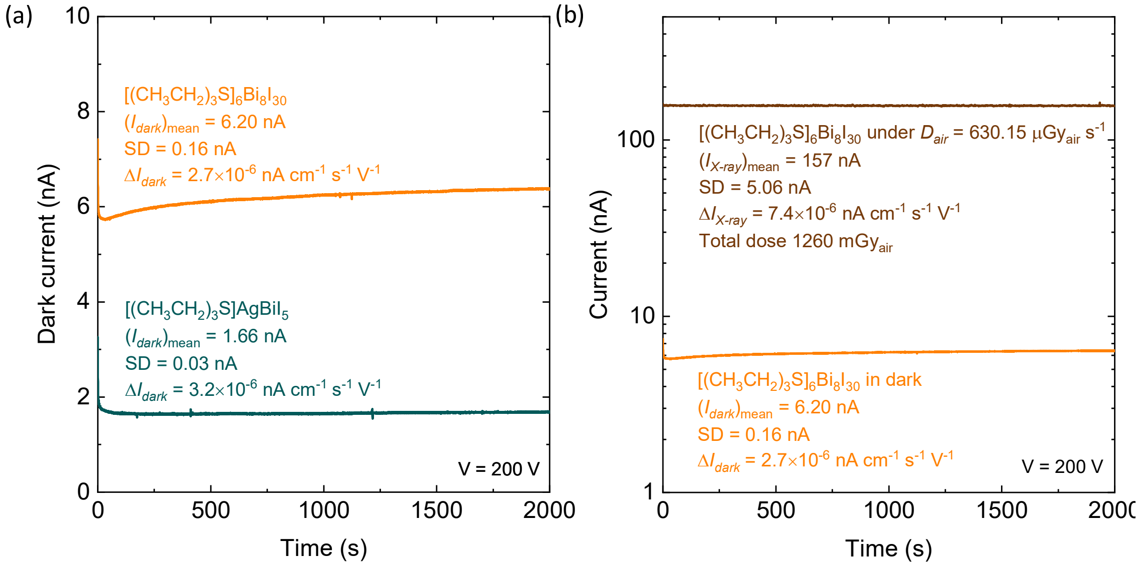

Fig. S13. Irradiation- and bias stability after six more month of storage of the $[(CH_3CH_2)_3S]AgBiI_5$- and $[(CH_3CH_2)_3S]_6Bi_8I_{30}$-based detectors. a) Dark current drift of the Au/$[(CH_3CH_2)_3S]_6Bi_8I_{30}$/Au and Au/$[(CH_3CH_2)_3S]AgBiI_5$/Au X-ray detectors under a bias voltage of 200 V during a measurement period of 2000 s. The dark current drift (ΔI), mean stabilized dark current (I_{dark}) and its standard deviation (SD) are also provided; b) The current response of the X-ray detector devices of $[(CH_3CH_2)_3S]_6Bi_8I_{30}$ and $[(CH_3CH_2)_3S]AgBiI_5$ under a 200 V bias voltage and 2000 s X-ray illumination (D_{air} = 630.15 µGy$_{air}$ s^{-1}).

Supplementary Note 7. Scanning electron microscopy images

A comparison of the microstructures of $[(CH_3CH_2)_3S]AgBiI_5$ and $[(CH_3CH_2)_3S]_6Bi_8I_{30}$ using scanning electron microscope (SEM) images at different levels of magnification is shown in Fig. S13 and S14. At lower magnifications (100X and 500X), both samples display smooth surfaces to a considerable extent. However, when observed at higher magnifications (2 000X and 4 000X), notable distinctions become apparent. $[(CH_3CH_2)_3S]AgBiI_5$ in Fig. S14 shows a consistent microstructure with fewer imperfections on its surface and less porosity, indicating a more uniform composition of materials. On the contrary, $[(CH_3CH_2)_3S]_6Bi_8I_{30}$ in Fig. S15 exhibits rougher surfaces with levels of porosity and more visible defects in its microstructure, suggesting variations in processing conditions or a more heterogeneous material structure.

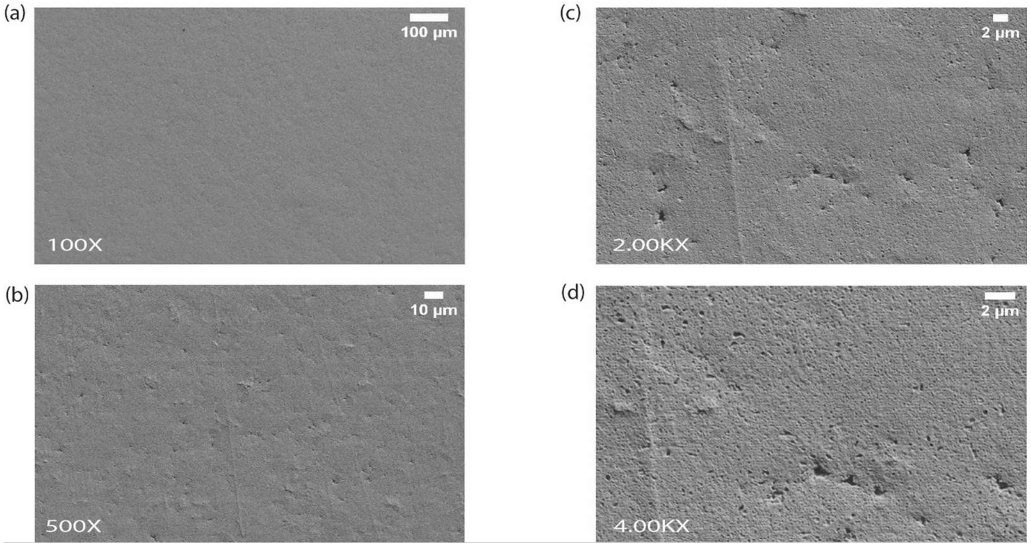

Fig. S14. Surface scanning electron microscopy (SEM) images of $[(CH_3CH_2)_3S]AgBiI_5$ at different magnifications. a) 100X, b) 500X, c) 2 000X, d) 4 000X.

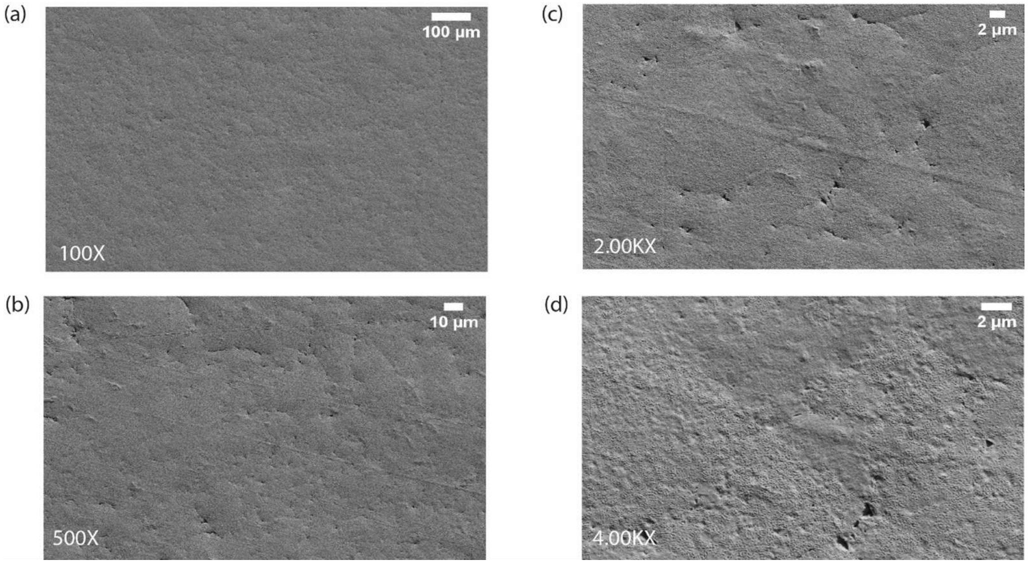

Fig. S15. Scanning electron microscopy (SEM) images of $[(CH_3CH_2)_3S]_6Bi_8I_{30}$ at different magnifications. a) 100X, b) 500X, c) 2 000X, d) 4 000X.

The cross-sectional scanning electron microscopy (SEM) images under various magnifications presented in Figures S16 and S17. These images allow close observation of the internal morphology and compactness of the pellets, in addition to the surface SEM images. The cross-sectional SEM images demonstrate that the pellets possess a homogeneously compressed morphology with no apparent large voids or cracks, which indicates the success of the isostatic compression process. Thus, we believe that the provided cross-sectional SEM characterization sufficiently demonstrates the compactness of the samples.

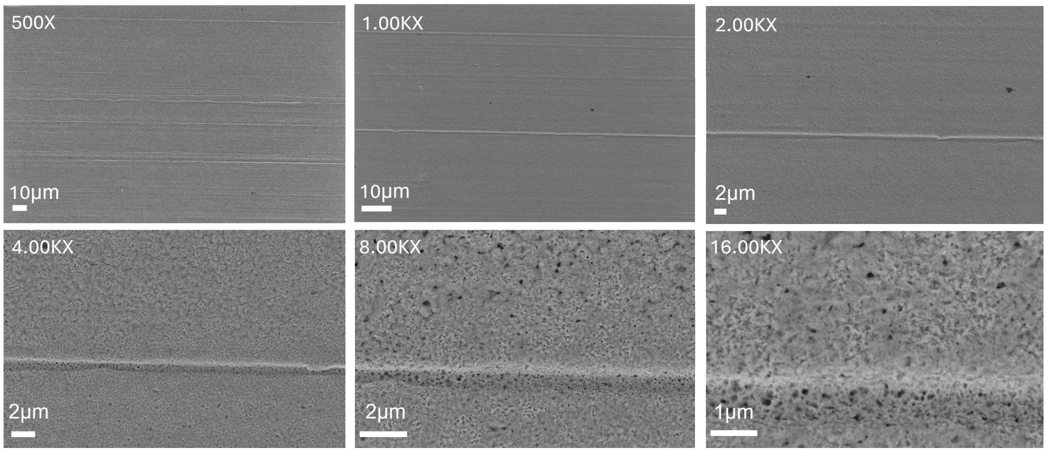

Fig. S16. Cross-sectional scanning electron microscopy (SEM) images of $[(CH_3CH_2)_3S]AgBiI_5$ at different magnifications.

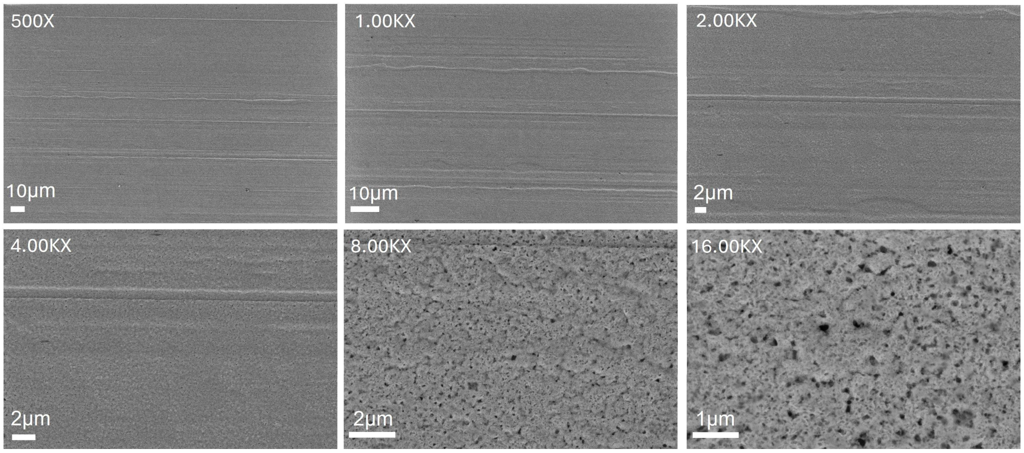

Fig. S17. Cross-sectional scanning electron microscopy (SEM) images of $[(CH_3CH_2)_3S]_6Bi_8I_{30}$ at different magnifications.

Supplementary Note 8. X-ray fluorescence data

Table S4. X-ray fluorescence (XRF) data for a pellet of $[(CH_3CH_2)_3S]AgBiI_5$.

**Element**	**Symbol**	**Theoretical Mass Percent (%)**	**XRF Measured Mass Percent (%)**	**Difference (%)**
Carbon	C	6.73	12.54	5.8
Sulfur	S	3	2.95	-0.04
Silver	Ag	10.08	11.2	1.12
Bismuth	Bi	19.52	22.53	3.01
Iodine	I	59.27	52.02	-7.24

Table S5. X-ray fluorescence (XRF) data for a pellet of $[(CH_3CH_2)_3S]_6Bi_8I_{30}$.

**Element**	**Symbol**	**Theoretical Mass Percent (%)**	**XRF Measured Mass Percent (%)**	**Difference (%)**
Carbon	C	6.98	15.08	8.1
Sulfur	S	3.11	3.72	0.61
Bismuth	Bi	26.99	30.31	3.32
Iodine	I	61.46	51.89	-9.57

References

[1] S. Shrestha, R. Fischer, G. J. Matt, P. Feldner, T. Michel, A. Osvet, I. Levchuk, B. Merle, S. Golkar, H. Chen, S. F. Tedde, O. Schmidt, R. Hock, M. Rührig, M. Göken, W. Heiss, G. Anton, C. J. Brabec, *Nat Photonics* **2017**, *11*, 436.

[2] B. Yang, W. Pan, H. Wu, G. Niu, J. H. Yuan, K. H. Xue, L. Yin, X. Du, X. S. Miao, X. Yang, Q. Xie, J. Tang, *Nat Commun* **2019**, *10*, 1989.

[3] W. Pan, B. Yang, G. Niu, K. H. Xue, X. Du, L. Yin, M. Zhang, H. Wu, X. S. Miao, J. Tang, *Advanced Materials* **2019**, *31*, 1904405.

[4] M. Hu, S. Jia, Y. Liu, J. Cui, Y. Zhang, H. Su, S. Cao, L. Mo, D. Chu, G. Zhao, K. Zhao, Z. Yang, S. F. Liu, *ACS Appl Mater Interfaces* **2020**, *12*, 16592.

[5] S. Tie, W. Zhao, D. Xin, M. Zhang, J. Long, Q. Chen, X. Zheng, J. Zhu, W. H. Zhang, *Advanced Materials* **2020**, *32*, 2001981.

[6] M. Daum, S. Deumel, M. Sytnyk, H. A. Afify, R. Hock, A. Eigen, B. Zhao, M. Halik, A. These, G. J. Matt, C. J. Brabec, S. F. Tedde, W. Heiss, *Adv Funct Mater* **2021**, *31*, 2102713.

[7] S. Deumel, A. van Breemen, G. Gelinck, B. Peeters, J. Maas, R. Verbeek, S. Shanmugam, H. Akkerman, E. Meulenkamp, J. E. Huerdler, M. Acharya, M. García-Batlle, O. Almora, A. Guerrero, G. Garcia-Belmonte, W. Heiss, O. Schmidt, S. F. Tedde, *Nat Electron* **2021**, *4*, 681.

[8] L. Yang, J. Pang, Z. Tan, Q. Xiao, T. Jin, J. Luo, G. Niu, J. Tang, *Frontiers of Optoelectronics* **2021**, *14*, 473.

[9] Y. Xiao, S. Jia, N. Bu, N. Li, Y. Liu, M. Liu, Z. Yang, S. Liu, *J Mater Chem A Mater* **2021**, *9*, 25603.

[10] S. Tie, D. Xin, S. Dong, B. Cai, J. Zhu, X. Zheng, *ACS Sustain Chem Eng* **2022**, *10*, 10743.

[11] S. Alghamdi, S. Bennett, C. Crean, J. Ghosh, H. Gibbard, R. Moss, J. Reiss, D. Wolfe, P. Sellin, *Applied Sciences (Switzerland)* **2022**, *12*, 2013.

[12] N. Bu, S. Jia, Y. Xiao, H. Li, N. Li, X. Liu, Z. Yang, K. Zhao, S. Liu, *J Mater Chem C Mater* **2022**, *10*, 6665.

[13] C. Zhang, *Detection* **2022**, *9*, 13.

[14] M. Li, H. Li, W. Li, B. Li, T. Lu, X. Feng, C. Guo, H. Zhang, H. Wei, B. Yang, *Advanced Materials* **2022**, *34*, 2108020.

[15] Y. Xiao, C. Xue, X. Wang, Y. Liu, Z. Yang, S. Liu, *ACS Appl Mater Interfaces* **2022**, *14*, 54867.

[16] W. Liu, T. Shi, J. Zhu, Z. Zhang, D. Li, X. He, X. Fan, L. Meng, J. Wang, R. He, Y. Ge, Y. Liu, P. K. Chu, X. F. Yu, *Advanced Science* **2023**, *10*, 2204512.

[17] T. Shi, W. Liu, J. Zhu, X. Fan, Z. Zhang, X. He, R. He, J. Wang, K. Chen, Y. Ge, X. Sun, Y. Liu, P. K. Chu, X. F. Yu, *Nano Res* **2023**, *16*, 9983.

[18] S. Jia, Y. Xiao, N. Bu, N. Li, D. Li, Z. Yang, S. Liu, *Adv Funct Mater* **2023**, *33*, 2213563.

[19] Y. Chai, Z. Juan, Y. Wu, Y. Liu, X. Li, *ACS Appl Electron Mater* **2023**, *5*, 544.

[20] Y. Ba, Y. Han, W. Zhu, T. Wang, J. Chi, H. Xi, T. Zhao, D. Chen, J. Zhang, C. Zhang, Y. Hao, *Chemical Engineering Journal* **2024**, *479*, 147726.

[21] W. Tress, *Journal of Physical Chemistry Letters* **2017**, *8*, 3106.

[22] E. A. Duijnstee, J. M. Ball, V. M. Le Corre, L. J. A. Koster, H. J. Snaith, J. Lim, *ACS Energy Lett* **2020**, *5*, 376.

[23] M. J. Berger, J. H. Hubbell, S. M. Seltzer, J. Chang, J. S. Coursey, R. Sukumar, D. S. Zucker, K. Olsen, **2010**, DOI 10.18434/T48G6X.

[24] A. Starkholm, Synthesis and Robotized Screening of Novel Perovskite Materials for Solar Cell Application, Doctoral Thesis, KTH Royal Institute of Technology, **2021**.

[25] Y. He, I. Hadar, M. G. Kanatzidis, *Nat Photonics* **2022**, *16*, 14.

[26] S. O. Kasap, *J. Phys. D: Appl. Phys* **2000**, *33*, 2853.

[27] K. R. Dudipala, T. H. Le, W. Nie, R. L. Z. Hoye, *Advanced Materials* **2024**, *36*, 2304523.

[28] C. A. Klein, *J Appl Phys* **1968**, *39*, 2029.
